# Triple‐Negative Breast Cancer Cells Resist Natural Killer Cell‐Mediated Killing Through Interleukin‐11 Trans‐Signaling

**DOI:** 10.1002/advs.202515772

**Published:** 2025-11-03

**Authors:** Hongmei Yang, Hao Jia, Renfei Wu, Haibo Tong, Liping Chen, Kathy Qian Luo

**Affiliations:** ^1^ Department of Biomedical Sciences Faculty of Health Sciences University of Macau Taipa Macao SAR 999078 China; ^2^ Ministry of Education Frontiers Science Center for Precision Oncology University of Macau Taipa Macao SAR 999078 China

**Keywords:** IL‐11, immune evasion, NK cell exhaustion, TNBC, trans‐signaling

## Abstract

Natural killer (NK) cell‐based therapies show great promise for treating triple‐negative breast cancer (TNBC). However, in the tumor microenvironment, some TNBC cells develop resistance to NK cell‐mediated killing and contribute to NK cell exhaustion. In this study, TNBC cell populations are isolated that can resist NK cell attacks and uncovered the underlying mechanisms. It is found that these resistant TNBC cells secrete high levels of interleukin‐11 (IL‐11). IL‐11 acts through a process known as trans‐signaling by forming complexes with the soluble IL‐11 receptor (sIL‐11R) and engaging the gp130 receptor on NK cells. This trans‐signaling activates the JAK1/STAT1/3 pathway, leading to the upregulation of p21 and subsequent cell cycle arrest in NK cells. As a result, the proliferation and IFNγ production of NK cells are inhibited, enabling TNBC cells to resist NK cell‐mediated killing. Disrupting IL‐11 or sIL‐11R in TNBC cells restores NK cell activity. Importantly, it is also found that IL‐11 expression is elevated in human TNBC tissues and negatively correlated with the number of NK cells in the tumor microenvironment. These findings identify IL‐11 trans‐signaling as a novel mechanism of immune evasion in TNBC and suggest that targeting this pathway may enhance the effectiveness of NK cell‐based therapies.

## Graphical abstract

Triple‐negative breast cancer cells express high levels of interleukin‐11 (IL‐11) that can form complexes with soluble IL‐11 receptor (sIL‐11R). These complexes can bind to gp130 on the membrane of natural killer (NK) cells through trans‐signaling to activate JAK1/STAT1/3/p21 downstream pathway. This activation arrests cell cycle progression of NK cells, decreasing their ability to attack breast cancer cells.

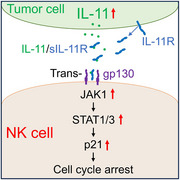

## 1 Introduction

Triple‐negative breast cancer (TNBC) makes up 15–20% of breast cancer cases. Compared to other subtypes, TNBC has a lower five‐year survival rate due to its aggressive nature, rapid progression, and poor prognosis.^[^ ^1^, ^2^ ^]^ TNBC lacks expression of estrogen and progesterone receptors and does not exhibit human epidermal growth factor receptor 2 amplification. Consequently, TNBC is not sensitive to either endocrine therapy or targeted therapy. Currently, chemotherapy is the primary treatment option; however, its effectiveness is limited, and many patients develop drug resistance.^[^ ^1^, ^3^ ^]^ In recent years, immunotherapy, particularly T cell and natural killer (NK) cell therapies, has gained significant attention. Unlike cytotoxic T cells, NK cells can directly recognize and kill tumor cells without tumor antigen presentation. NK cell therapy offers several advantages over T cell therapy, such as a broader range of cell sources, a better safety profile, and off‐the‐shelf availability.^[^ ^4^, ^5^ ^]^ However, within the tumor microenvironment (TME), some TNBC cells can resist NK cell‐mediated killing and induce NK cell exhaustion, which can lead to the advancement of tumors.^[^ ^6^, ^7^ ^]^ Understanding how TNBC cells resist NK cell‐mediated killing is essential for developing effective NK cell‐based therapies for TNBC patients.

Several studies have explored how TNBC cells resist NK cell‐mediated killing. Research indicates that NK cells in the TME express PD‐1, while TNBC cells inhibit the cytotoxicity of NK cells through the PD‐L1/PD‐1 axis. Although immunotherapies that block PD‐1 and PD‐L1 have been widely used in TNBC patients, their therapeutic effects have been quite limited.^[^ ^8^, ^9^, ^10^, ^11^, ^12^ ^]^ TGFβ plays a significant immunosuppressive role in the TME. Studies have shown that TGFβ can inhibit glycolysis and oxidative phosphorylation in NK cells within both TNBC and non‐TNBC TMEs, leading to NK cell dysfunction.^[^ ^13^, ^14^ ^]^ Hypoxia is a crucial characteristic of the TME. Hypoxia‐inducible factor‐1α suppresses the function of T cells and NK cells through epigenetic regulation.^[^ ^15^ ^]^ After TNBC cells migrate to the liver, NK cells in that region help maintain these TNBC cells in a dormant state by releasing interferon gamma (IFNγ). Hepatic stellate cells produce CXCL12, which binds to CXCR4 receptors on the surface of NK cells. This interaction inhibits the proliferation of NK cells and the release of IFNγ, facilitating the growth of dormant TNBC cells.^[^ ^16^ ^]^ The aspartic protease cathepsin D (cath‐D) is an extracellular protein produced by tumor cells and is associated with poor prognosis in TNBC patients. Studies have found that cath‐D can inhibit NK cell‐mediated killing in the TME and promote tumor immune escape. Antibodies targeting cath‐D have been generated to enhance NK cell activity and improve the effectiveness of chemotherapy for TNBC patients.^[^ ^17^, ^18^ ^]^

Despite existing studies, the mechanisms by which TNBC cells resist NK cell‐mediated killing and induce NK cell exhaustion are not fully understood. Moreover, the heterogeneity of TNBC cells allows them to employ various strategies to evade NK cell attacks. In the present study, we utilized a 3D coculture system of TNBC and NK cells to identify the TNBC cells capable of resisting NK cell‐mediated killing. RNA sequencing analysis revealed that these resistant TNBC cells expressed high levels of interleukin‐11 (IL‐11). Further mechanism studies demonstrated that IL‐11 activated the JAK1/STAT1/3 signaling pathway in NK cells through trans‐signaling. This activation resulted in the upregulation of p21, leading to cell cycle arrest in NK cells. Consequently, the proliferation and IFNγ production of NK cells were inhibited, allowing TNBC cells to resist NK cell‐mediated killing and form larger tumors in orthotopic mouse models. More importantly, analysis of clinical samples showed that expression levels of IL‐11 in TNBC tissues were significantly higher than those in normal tissues, and IL‐11 expression was negatively correlated with the number of NK cells in the TME. Our study reveals a novel mechanism by which TNBC cells resist NK cell‐mediated killing. Furthermore, our findings suggest that targeting the IL‐11 trans‐signaling pathway with antibodies or small‐molecule inhibitors may be a promising approach to enhance the effectiveness of NK cell‐based therapies.

## 2 Results

### 2.1 G10 Cells Resist NK Cell‐Mediated Killing In Vitro and In Vivo

**Figure 1:**
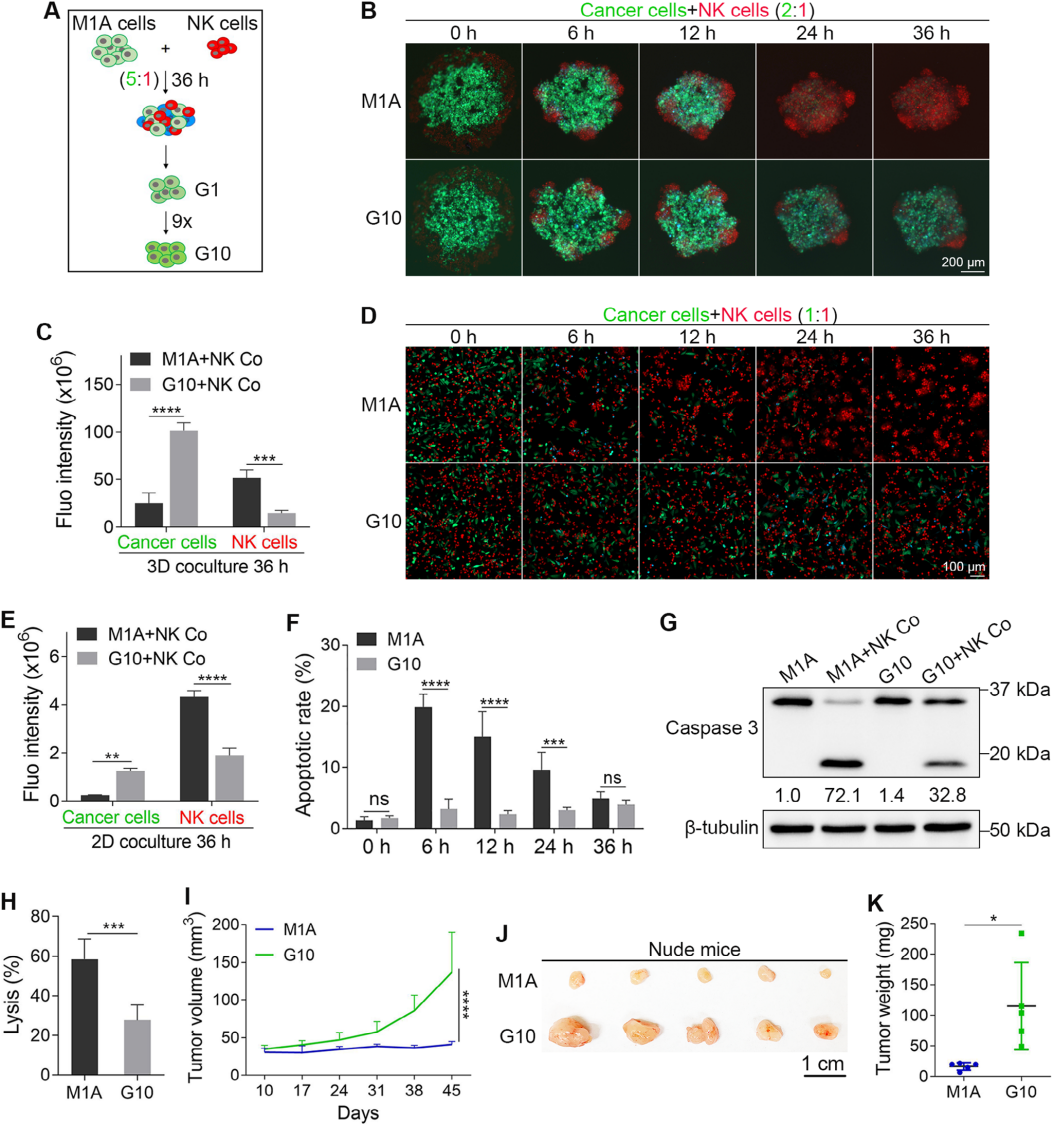
G10 cells resist NK cell‐mediated killing in vitro and in vivo. A) The scheme shows how G10 cells were generated. B) Fluorescent images show the coculture of M1A or G10 cells with NK cells under 3D conditions. C) Quantitative results show the total green and red fluorescence intensities after 36 h of coculture under 3D conditions (*n* = 4). D) Fluorescent images show the coculture of M1A or G10 cells with NK cells under 2D conditions. E) Quantitative results show the total green and red fluorescence intensities after 36 h of coculture under 2D conditions (*n* = 4). F) Apoptotic rates of M1A and G10 cells during coculture with NK cells under 2D conditions. The apoptotic rates were measured using FRET imaging (*n* = 4). G) WB shows the caspase 3 cleavage when M1A or G10 cells were cocultured with NK cells under 2D conditions for 12 h (*n* = 3). H) A calcein‐AM release assay shows the lysis rates of M1A and G10 cells when cocultured with NK cells under 2D conditions for 6 h (*n* = 5). I) The tumor growth of M1A and G10 cells, which were inoculated into the mammary fat pads of Nude mice (*n* = 5). J) The images of dissected tumors. K) Quantitative results show the tumor weights (*n* = 5). The sizes of scale bars are indicated in each image. The data were presented as mean ± SD. Statistical significance was determined by two‐way ANOVA (C, E, F, I) or *t*‐test (H, K). ^*^ *p* < 0.05, ^**^ *p* < 0.01, ^***^ *p* < 0.001, ^****^ *p* < 0.0001, ns: not significant.

To screen TNBC cells that can resist NK cell‐mediated killing, we injected MDA‐MB‐231 cells labeled with a fluorescence resonance energy transfer (FRET)‐based apoptotic biosensor^[^ ^19^, ^20^, ^21^, ^22^ ^]^ into the tail vein of Nude mice, which have normal NK cell activity but impaired T cell activity^[^ ^23^, ^24^ ^]^ and isolated metastatic lung tumors for two rounds. The tumors were dissected and digested with collagenase to collect the cancer cells. Finally, we got a stable cell line, named M1A.^[^ ^25^ ^]^ To determine whether M1A cells could resist NK cell‐mediated killing, we cocultured them with red fluorescent protein tdTomato‐labeled NK‐92MI (NK‐92MI‐tdT) cells in vitro at ratios of 5:1, 2:1, and 1:1 under 3D conditions for 36 h. The FRET‐based apoptotic biosensor emits green fluorescence when cells are alive and blue fluorescence when they are undergoing apoptosis. Thus, we could easily detect the survival of M1A cells by FRET imaging. The results showed that some M1A cells could resist NK cell‐mediated killing at the ratio of 5:1, while almost all M1A cells were killed by NK cells at the ratios of 2:1 and 1:1 (Figure S1, Supporting Information). The surviving M1A cells were collected and named G1. To further select cancer cells that could resist NK cell‐mediated killing, we repeated the coculture process nine more times at the ratio of 5:1, eventually obtaining a cell line called G10 (**Figure** **1**A).

We then cocultured G10 or M1A cells with NK‐92MI‐tdT cells at a ratio of 2:1 and conducted FRET imaging analysis. The results showed that after 36 h of coculture, almost all M1A cells were killed by NK cells, whereas a significant number of G10 cells remained green. Interestingly, the red fluorescence of NK cells in the G10 group was 3.3 times weaker than that in the M1A group (Figure 1B,C). These data indicated that G10 cells had a greater ability to resist NK cell‐mediated killing and simultaneously inhibit the proliferation of NK cells. Next, we compared the survival ability of G10 and M1A cells when exposed to NK cells at a ratio of 1:1 under 2D conditions and observed similar results (Figure 1D,E). Further analysis revealed that the apoptotic rates of M1A cells at 6, 12, and 24 h of 2D coculture were 6.2 times, 6.3 times, and 3.2 times higher than those of G10 cells (Figure 1F). Additionally, Western blotting (WB) analysis showed that after 12 h of 2D coculture, the intensity of cleaved caspase‐3 in M1A cells was 2.2 times stronger than that in G10 cells (Figure 1G). In addition to FRET imaging, we employed the conventional calcein‐AM release assay to assess the death of cancer cells following NK cell exposure. The results showed that after 6 h of coculture under 2D conditions, the lysis rate of M1A cells was 2.1 times higher than that of G10 cells (Figure 1H).

Next, we injected M1A or G10 cells into the mammary fat pads of Nude mice to evaluate their ability to form tumors in vivo. At the end of the experiment, the tumor volumes in the G10 group were 3.3 times larger than those in the M1A group (Figure 1I). Then, the tumors were dissected, and the results showed that tumor weights of G10 cells were 7.0 times greater than those of M1A cells (Figure 1J,K).

### 2.2 G10 Cells Inhibit the Proliferation and IFNγ Production of NK Cells

**Figure 2:**
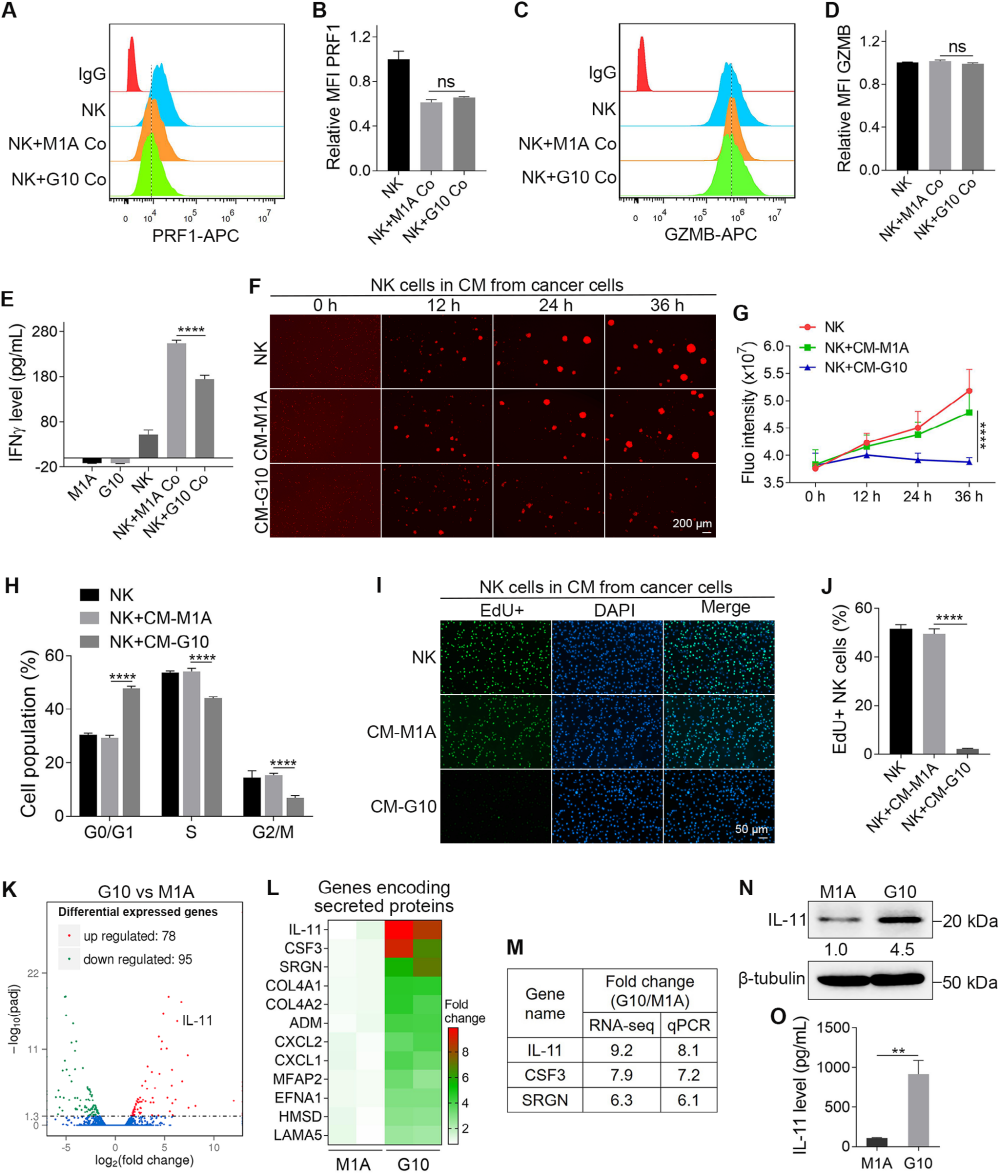
G10 cells inhibit the proliferation and IFNγ production of NK cells. A–D) Flow cytometry analysis of PRF1 and GZMB expression in NK cells cocultured with M1A or G10 cells for 24 h (*n* = 3). E) The concentrations of IFNγ in the monoculture or coculture media after 24 h of culture (*n* = 3). F) Fluorescent images show the proliferation of NK cells in normal NK cell culture medium or CM from M1A or G10 cells. G) Quantified results show the total fluorescence intensities of NK cells in (F) (*n* = 4). H) The cell cycle distribution of NK cells after being cultured for 24 h in normal NK cell culture medium or CM from M1A or G10 cells (*n* = 3). I) Fluorescent images show the EdU‐positive NK cells after being cultured for 24 h in normal NK cell culture medium or CM from M1A or G10 cells. J) Quantified results show the percentages of EdU‐positive NK cells (*n* = 3). K) RNA‐seq analysis shows differentially expressed genes between M1A and G10 cells. L) The top 12 genes encoding secreted proteins upregulated in G10 cells compared to M1A cells. M) The fold changes of IL‐11, CSF3, and SRGN in RNA‐seq and qPCR (*n* = 3). N) WB shows the upregulation of IL‐11 in G10 cells (*n* = 3). O) ELISA results show there is more IL‐11 in the CM from G10 cells (*n* = 3). The sizes of scale bars are indicated in each image. The data were presented as mean ± SD. Statistical significance was determined by one‐way ANONA (B, D, E, J), two‐way ANOVA (G, H), or *t*‐test (O). ^**^ *p* < 0.01, ^****^ *p* < 0.0001, ns: not significant.

Using flow cytometry, we measured the levels of perforin 1 (PRF1) and granzyme B (GZMB) in NK cells after coculturing them with M1A or G10 cells for 24 h. The results indicated that the secretion of PRF1 and GZMB by NK cells was similar between the M1A and G10 coculture groups (**Figure** **2**A–D). However, enzyme‐linked immunosorbent assay (ELISA) data revealed that G10 cells reduced the total level of IFNγ in the coculture medium by 31.4% after 24 h compared to M1A cells (Figure 2E). This decrease in IFNγ levels may be attributed to the reduced proliferation of NK cells (Figure 1B–E).

We hypothesized that G10 cells secrete factors that inhibit the proliferation of NK cells. To test this, we treated NK cells with conditioned medium (CM) from G10 and M1A cells. The results showed that NK cells grew normally in the CM from M1A cells (CM‐M1A). In contrast, the fluorescence intensity of NK cells barely increased after 36 h of culture in the CM from G10 (CM‐G10) (Figure 2F,G). Next, we analyzed the cell cycle distribution of NK cells after 24 h of culture in either CM‐M1A or CM‐G10. Compared to the control NK cells, those cultured in CM‐G10 exhibited a 17.4% increase in the G0/G1 phase, while the percentages of cells in the S and G2/M phases decreased by 9.6% and 7.6%, respectively. In comparison, the cell cycle distribution of NK cells cultured in CM‐M1A remained largely unchanged (Figure 2H; Figure S2, Supporting Information). Additionally, an EdU incorporation assay was conducted to assess the DNA synthesis of NK cells cultured in different media for 24 h. The results revealed that 51.5% of NK cells in the control group and 49.5% in the CM‐M1A group were EdU‐positive, while only 2.1% of NK cells in the CM‐G10 group were EdU‐positive (Figure 2I,J). All these data indicated that G10 cells inhibited the proliferation of NK cells through the secretion of specific factors.

To identify the relevant factors, we conducted RNA sequencing (RNA‐seq) analysis on M1A and G10 cells. The results indicated that a total of 173 genes were differentially expressed; among these, 78 genes were significantly upregulated, and 95 genes were downregulated in G10 cells compared to M1A cells (Figure 2K). Among these 78 upregulated genes, we then focused on the top 12 genes encoding secreted proteins and found that IL‐11 was the most highly expressed (Figure 2L). The top three upregulated genes, including IL‐11, CSF3, and SRGN, were further validated through quantitative polymerase chain reaction (qPCR) analysis (Figure 2M). WB analysis showed that the protein level of IL‐11 was 4.5‐fold upregulated in G10 cells (Figure 2N). More importantly, ELISA data showed that the concentration of IL‐11 in the CM from G10 cells was 8.7‐fold upregulated (Figure 2O).

### 2.3 G10 Cells Become Sensitive to NK Cell‐Mediated Killing, and They Cannot Inhibit the Proliferation and IFNγ Production of NK Cells after IL‐11 Knockdown

**Figure 3:**
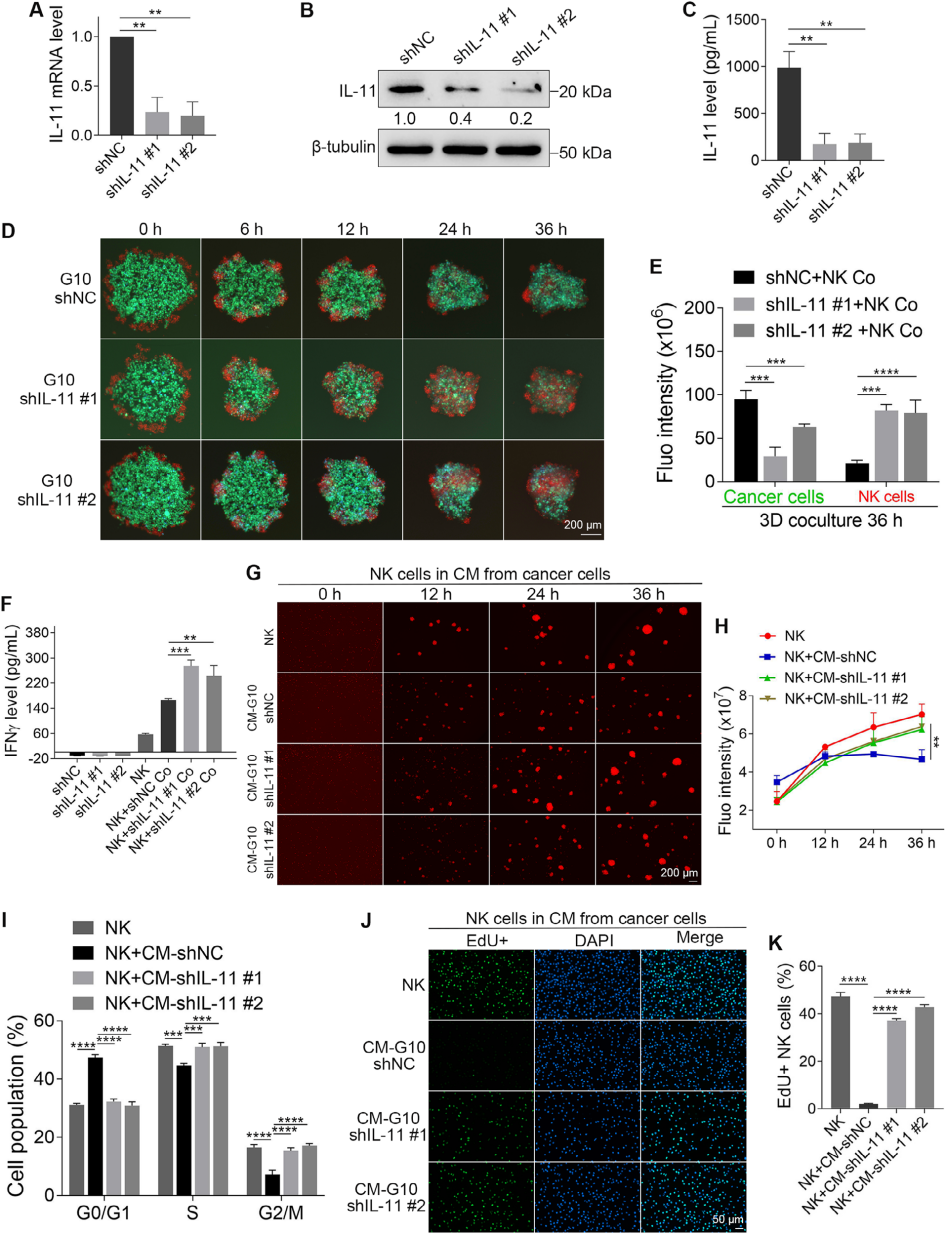
G10 cells became sensitive to NK cell‐mediated killing, and they could not inhibit the proliferation and IFNγ production of NK cells after IL‐11 knockdown. A,B) The knockdown of IL‐11 in G10 cells was confirmed by qPCR and WB (*n* = 3). C) ELISA assays show the reduced IL‐11 in the CM from IL‐11‐knockdown G10 cells (*n* = 3). D) Fluorescent images show the coculture of IL‐11‐knockdown G10 cells with NK cells at the ratio of 2:1. E) Quantified results show the total green and red fluorescence intensities after 36 h of coculture in (D) (*n* = 4). F) The concentrations of IFNγ in the monoculture or coculture media after 24 h of culture (*n* = 3). G) Fluorescent images show the proliferation of NK cells cultured in CM from IL‐11‐knockdown G10 cells. H) Quantified results show the total fluorescence intensities of NK cells in (G) (*n* = 3). I) The cell cycle distribution of NK cells after being cultured for 24 h in CM from IL‐11‐knockdown G10 cells (*n* = 3). J) Fluorescent images show the EdU‐positive NK cells after being cultured for 24 h in CM from IL‐11‐knockdown G10 cells. K) Quantified results show the percentages of EdU‐positive NK cells (*n* = 3). The sizes of scale bars are indicated in each image. The data were presented as mean ± SD. Statistical significance was determined by one‐way ANONA (A, C, F, K) or two‐way ANOVA (E, H, I). ^**^ *p* < 0.01, ^***^ *p* < 0.001, ^****^ *p* < 0.0001.

The expression of IL‐11 in G10 cells was inhibited using short hairpin RNA (shRNA)‐mediated knockdown. Results from qPCR and WB demonstrated that both IL‐11 targeting shRNAs (shIL‐11) significantly reduced the mRNA and protein levels of IL‐11 in G10 cells compared to the non‐targeting negative control shRNAs (shNC) (**Figure** **3**A,B). Importantly, the ELISA data indicated that both shRNAs reduced the concentrations of IL‐11 in the CM from G10 cells by 82.7% and 81.1%, respectively (Figure 3C). After the knockdown of IL‐11, G10 cells exhibited increased sensitivity to NK cell‐mediated killing, and NK cells grew better (Figure 3D). Statistical data revealed that the red fluorescence of NK cells in the knockdown group was 3.8 times higher than that of the control group (Figure 3E). In addition, NK cells secreted 1.6 times more IFNγ when cultured with IL‐11‐knockdown G10 cells (Figure 3F).

More importantly, after the knockdown of IL‐11 in G10 cells, the CM from G10 cells could not suppress the proliferation of NK cells (Figure 3G,H). Additionally, flow cytometry analysis indicated that the inhibitory effects on the NK cell cycle were reversed after the IL‐11 knockdown in G10 cells (Figure 3I; Figure S3, Supporting Information). EdU staining results showed that the CM from G10 cells could no longer inhibit DNA synthesis in NK cells following the IL‐11 knockdown (Figure 3J,K).

### 2.4 IL‐11 Can also Help 231‐LC Cells Resist NK Cell‐Mediated Killing by Inhibiting the Proliferation and IFNγ Production of NK Cells

**Figure 4:**
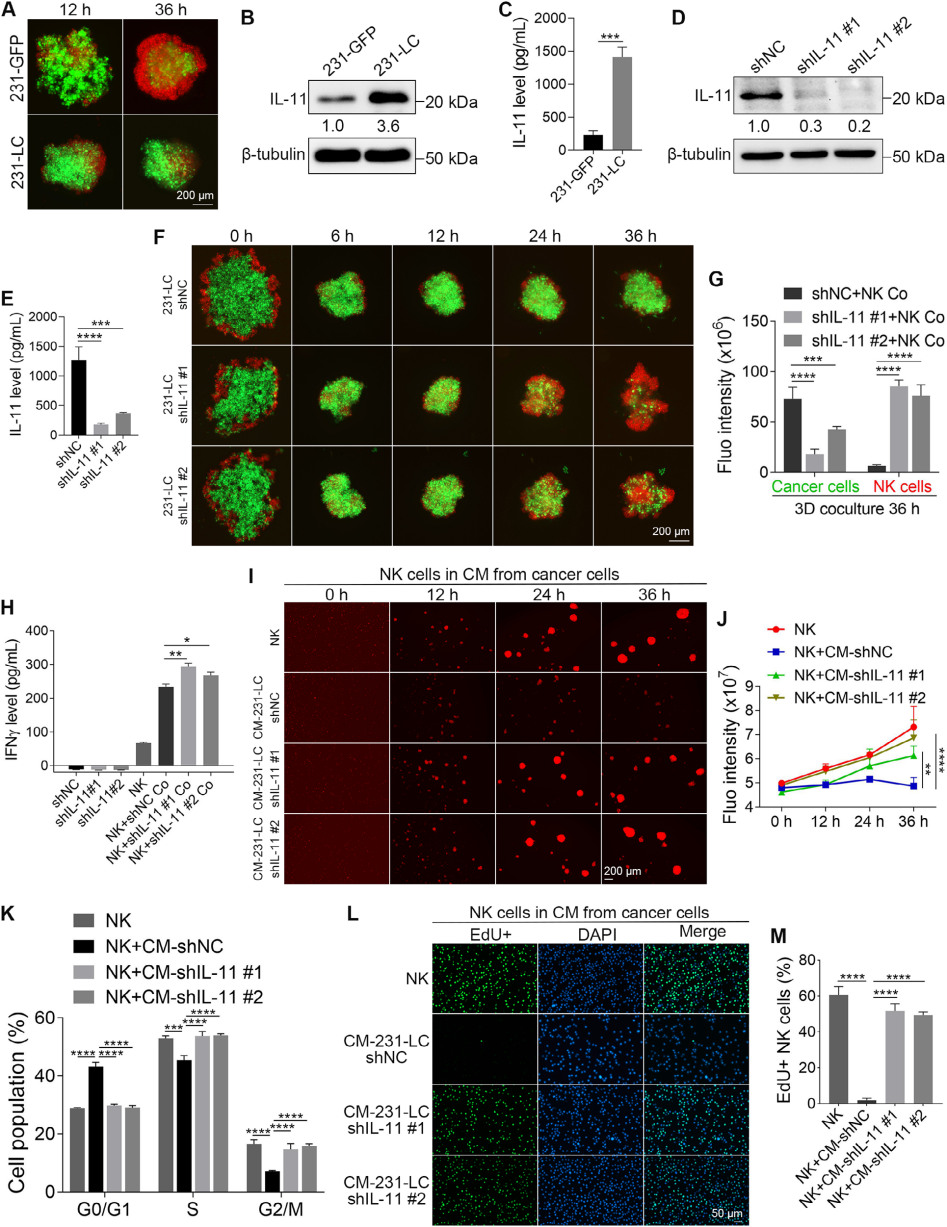
IL‐11 helps 231‐LC cells resist NK cell‐mediated killing by inhibiting NK cell proliferation and IFNγ production. A) Fluorescent images show the coculture of 231‐GFP or 231‐LC cells with NK cells. B) WB shows the upregulation of IL‐11 in 231‐LC cells (*n* = 3). C) ELISA results show there is more IL‐11 in the CM from 231‐LC cells (*n* = 3). D) The knockdown of IL‐11 in 231‐LC cells was confirmed by WB (*n* = 3). E) ELISA assays show the reduced IL‐11 in the CM from IL‐11 knockdown 231‐LC cells (*n* = 3). F) Fluorescent images show the coculture of IL‐11‐knockdown 231‐LC cells with NK cells. G) Quantified results show the total green and red fluorescence intensities after 36 h of coculture in (F) (*n* = 3). H) The concentrations of IFNγ in the monoculture or coculture media after 24 h of culture (*n* = 3). I) Fluorescent images show the proliferation of NK cells cultured in CM from IL‐11‐knockdown 231‐LC cells. J) Quantified results show the total fluorescence intensities of NK cells in (I) (*n* = 3). K) The cell cycle distribution of NK cells after being cultured for 24 h in CM from IL‐11‐knockdown 231‐LC cells (*n* = 3). L) Fluorescent images show the EdU‐positive NK cells after being cultured for 24 h in CM from IL‐11‐knockdown 231‐LC cells. M) Quantified results show the percentages of EdU‐positive NK cells (*n* = 3). The sizes of scale bars are indicated in each image. The data were presented as mean ± SD. Statistical significance was determined by *t*‐test (C), one‐way ANONA (E, H, M), or two‐way ANOVA (G, J, K). ^*^ *p* < 0.05, ^**^ *p* < 0.01, ^***^ *p* < 0.001, ^****^ *p* < 0.0001.

We have demonstrated that G10 cells can resist NK cell‐mediated killing by upregulating IL‐11. To eliminate the potential effects of clonal heterogeneity and the apoptotic biosensor, we generated GFP‐labeled MDA‐MB‐231 (231‐GFP) cells and injected them into the tail vein of Nude mice to obtain new lung colony cells (231‐LC). We then cocultured 231‐GFP or 231‐LC cells with NK cells at a ratio of 2:1 for 36 h. The results indicated that most 231‐GFP cells were killed by NK cells, while 231‐LC cells displayed resistance to NK cell‐mediated killing. At the same time, the proliferation of NK cells was inhibited by 231‐LC cells (**Figure** **4**A). Since 231‐LC cells already exhibited a strong ability to resist NK cell‐mediated killing, we did not conduct further in vitro coculture selections to create a cell line like G10 cells. We wanted to determine whether the resistance of 231‐LC cells to NK cell‐mediated killing was due to the upregulation of IL‐11. First, qPCR and WB results revealed that 231‐LC cells expressed significantly higher levels of IL‐11 compared to 231‐GFP cells (Figure 4B; Figure S4A, Supporting Information). More importantly, the concentration of IL‐11 in the CM from 231‐LC cells was 6.1‐fold upregulated (Figure 4C).

In the next step, shRNAs were used to reduce IL‐11 levels in 231‐LC cells, and both shRNAs demonstrated effective knockdown, as confirmed by qPCR and WB analysis (Figure 4D; Figure S4B, Supporting Information). ELISA results indicated that after the knockdown of IL‐11 in 231‐LC cells, IL‐11 levels in the CM decreased by 78.5% (Figure 4E). Coculture experiments revealed that IL‐11‐knockdown 231‐LC cells became more sensitive to NK cell‐mediated killing and were unable to inhibit NK cell proliferation (Figure 4F,G). Additionally, when NK cells were cocultured with IL‐11‐knockdown 231‐LC cells, the levels of IFNγ in the coculture media increased by 20.1% (Figure 4H).

Similar to the IL‐11‐knockdown G10 cells, the CM from IL‐11‐knockdown 231‐LC cells could not inhibit the proliferation of NK cells (Figure 4I,J). The cell cycle of NK cells was blocked at the G0/G1 phase when treated with the CM from shNC 231‐LC cells. This cell cycle block could be reversed after the knockdown of IL‐11 (Figure 4K; Figure S4C, Supporting Information). EdU staining demonstrated that the CM from the 231‐LC cells gradually inhibited DNA synthesis in NK cells (Figure S4D,E, Supporting Information). After the knockdown of IL‐11 in 231‐LC cells, the CM no longer inhibited NK cell DNA synthesis (Figure 4L,M). Although the NK cell cycle was blocked and DNA synthesis was inhibited, Annexin V staining results indicated that NK cells did not undergo apoptosis following CM treatment (Figure S4F,G, Supporting Information). It is important to note that we do not rule out the possibility that NK cells may eventually undergo apoptosis if the coculture period is extended.

**Figure 5:**
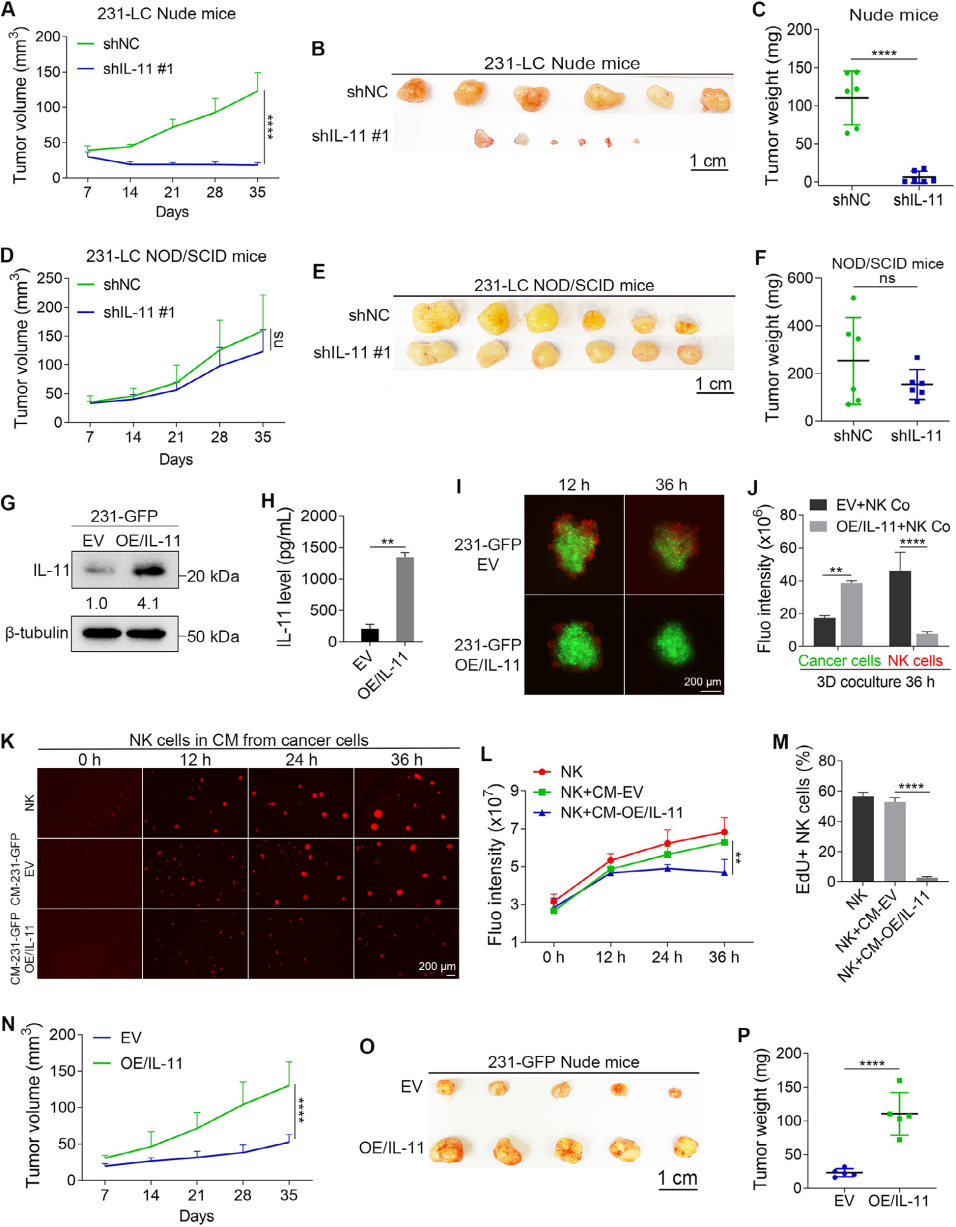
IL‐11 overexpressing 231‐GFP cells gain the ability to resist NK cell‐mediated killing and inhibit the proliferation and IFNγ production of NK cells. A–C) The tumor volumes, dissected tumors, and tumor weights of shNC 231‐LC cells and IL‐11‐knockdown 231‐LC cells in Nude mice (*n* = 6). D–F) The tumor volumes, dissected tumors, and tumor weights of shNC 231‐LC cells and IL‐11‐knockdown 231‐LC cells in NOD/SCID mice (*n* = 6). G) The overexpression of IL‐11 in 231‐GFP cells was confirmed by WB (*n* = 3). H) ELISA assays show the increased IL‐11 in the CM from IL‐11 overexpressing 231‐GFP cells (*n* = 3). I) Fluorescent images show the coculture of IL‐11 overexpressing 231‐GFP cells with NK cells at the ratio of 2:1. J) Quantified results show the total green and red fluorescence intensities after 36 h of coculture in (I) (*n* = 3). K) Fluorescent images show the proliferation of NK cells cultured in CM from IL‐11 overexpressing 231‐GFP cells. L) Quantified results show the total fluorescence intensities of NK cells in (K) (*n* = 3). M) Quantified results show the percentages of EdU‐positive NK cells after being cultured for 24 h in CM from IL‐11 overexpressing 231‐GFP cells (*n* = 3). N) The tumor volumes of control and IL‐11 overexpressing 231‐GFP cells (*n* = 5). O) The images of dissected tumors. P) Quantitative results show the tumor weights (*n* = 5). The sizes of scale bars are indicated in each image. The data were presented as mean ± SD. Statistical significance was determined by *t*‐test (C, F, H, P), one‐way ANONA (M), or two‐way ANOVA (A, D, J, L, N). ^**^ *p* < 0.01, ^****^ *p* < 0.0001, ns: not significant.

To investigate whether 231‐LC cells resist NK cell‐mediated killing through the upregulation of IL‐11 in vivo, we injected either shNC 231‐LC cells or IL‐11‐knockdown 231‐LC cells into the mammary fat pads of Nude or NOD/SCID mice. Nude mice possess normal NK cells, whereas the NK cell activity in NOD/SCID mice is severely compromised.^[^ ^26^, ^27^, ^28^ ^]^ The knockdown of IL‐11 in 231‐LC cells resulted in a significant decrease in tumor growth in Nude mice (**Figure** **5**A). Statistical data showed that the tumor weights decreased by 94.1% after IL‐11 knockdown (Figure 5B,C). Conversely, IL‐11 knockdown did not affect tumor growth in NOD/SCID mice (Figure 5D–F). These findings indicated that IL‐11 promotes tumor growth in vivo by enhancing the resistance of 231‐LC cells to NK cell‐mediated killing.

To further investigate the role of IL‐11 in helping cancer cells resist NK cell‐mediated killing, we overexpressed IL‐11 in 231‐GFP cells (Figure 5G; Figure S5A, Supporting Information). ELISA assays indicated that the IL‐11 concentration in the CM from IL‐11 overexpressing 231‐GFP cells was 6.4‐fold upregulated (Figure 5H). The coculture results demonstrated that after IL‐11 overexpression, the 231‐GFP cells were able to resist NK cell‐mediated killing and inhibit the proliferation of NK cells (Figure 5I,J). Moreover, when NK cells were cocultured with IL‐11 overexpressing 231‐GFP cells, the levels of IFNγ in the coculture media were reduced by 35.1% compared to the control 231‐GFP cells (Figure S5B, Supporting Information). The CM from IL‐11 overexpressing 231‐GFP cells also exhibited the ability to inhibit NK cell proliferation (Figure 5K,L). EdU staining confirmed that the DNA synthesis of NK cells was inhibited by the CM from IL‐11 overexpressing 231‐GFP cells (Figure 5M). In an orthotopic tumor model, IL‐11 overexpressing 231‐GFP cells grew more rapidly and formed tumors that were 4.8 times larger in Nude mice (Figure 5N–P).

### 2.5 IL‐11 Inhibits the Proliferation of NK Cells Through Trans‐Signaling

**Figure 6:**
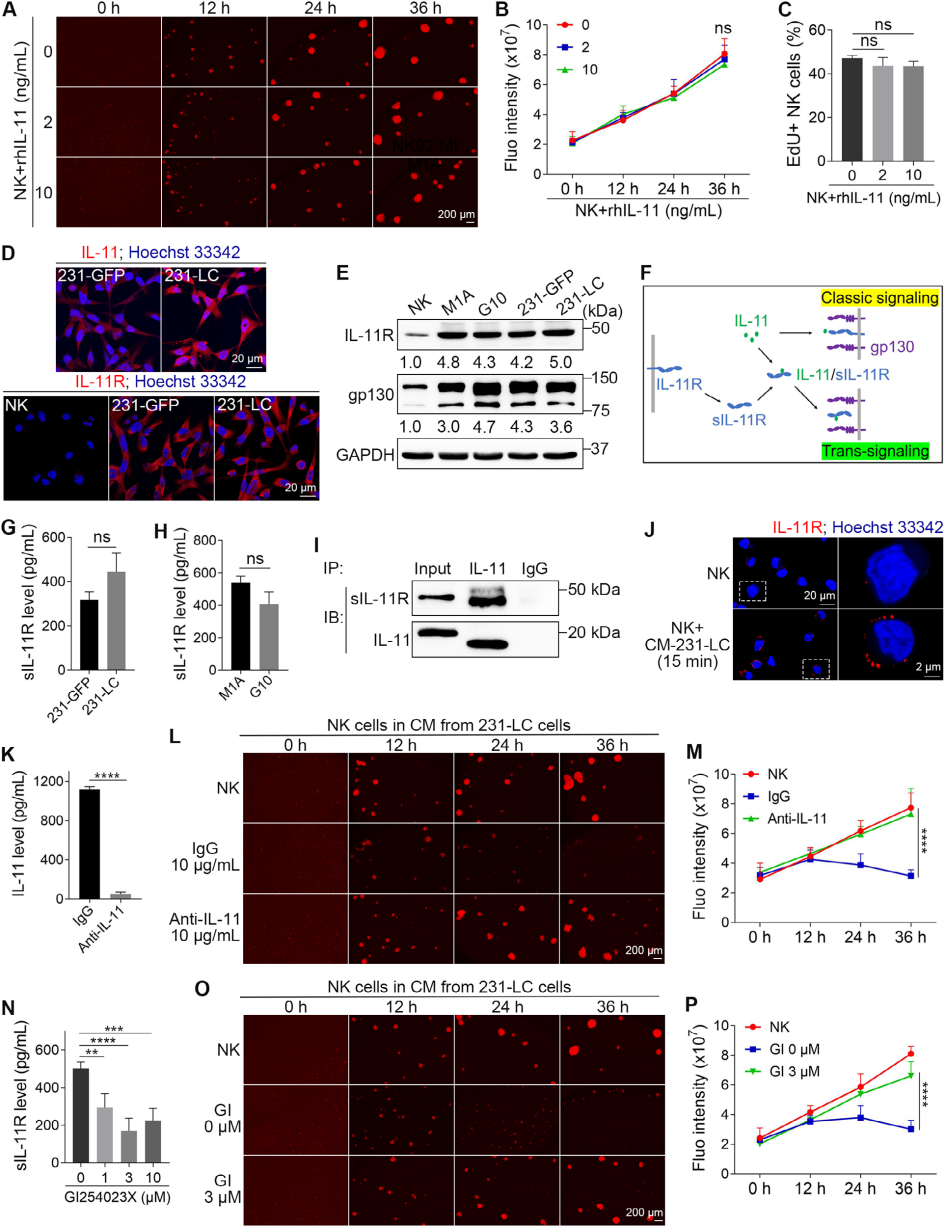
IL‐11 inhibits the proliferation of NK cells through trans‐signaling. A) Fluorescent images show the proliferation of NK cells upon rhIL‐11 treatment. B) Quantified results show the total fluorescence intensities of NK cells in (A) (*n* = 4). C) Quantified results show the percentages of EdU‐positive NK cells after 24 h treatment of rhIL‐11 (*n* = 4). D) Immunostaining shows the expression levels of IL‐11 and IL‐11R. E) WB shows the protein levels of IL‐11R and gp130 in NK and cancer cells (*n* = 3). F) A diagram shows the classic signaling and trans‐signaling of IL‐11. G, H) ELISA assays show the levels of sIL‐11R in the CM from cancer cells (*n* = 3). I) Co‐IP assay shows the binding of IL‐11 and sIL‐11R in the CM from 231‐LC cells (*n* = 3). J) Immunostaining shows the enrichment of IL‐11R signals on the surface of NK cells. K) The levels of IL‐11 in the CM from 231‐LC cells after neutralization treatment (*n* = 4). L) Fluorescent images show the proliferation of NK cells cultured in IL‐11 neutralized CM from 231‐LC cells. M) Quantified results show the total fluorescence intensities of NK cells in (L) (*n* = 4). N) The levels of sIL‐11R in the CM from 231‐LC cells with the ADAM10 inhibitor, GI254023X (*n* = 4). O) Fluorescent images show the proliferation of NK cells cultured in CM from 231‐LC cells with GI254023X. P) Quantified results show the total fluorescence intensities of NK cells in (O) (*n* = 4). The sizes of scale bars are indicated in each image. The data were presented as mean ± SD. Statistical significance was determined by two‐way ANOVA (B, M, P), one‐way ANONA (C, N), or *t*‐test (G, H, K). ^**^ *p* < 0.01, ^***^ *p* < 0.001, ^****^ *p* < 0.0001, ns: not significant.

To investigate how IL‐11 inhibits the proliferation of NK cells, we cultured NK cells in normal culture medium supplemented with recombinant human IL‐11 proteins (rhIL‐11). Interestingly, even at a concentration of 10 ng mL^−1^, rhIL‐11 could not inhibit the proliferation of NK cells (**Figure** **6**A,B). EdU staining analysis further indicated that the DNA synthesis in NK cells was also unaffected by rhIL‐11 (Figure 6C). These results suggested that NK cells might lack IL‐11 receptors (IL‐11R). To explore this hypothesis, we conducted immunostaining experiments on NK cells and cancer cells. The results revealed that 231‐LC cells expressed significantly higher levels of IL‐11 compared to 231‐GFP cells; however, both cell types exhibited substantial expression of IL‐11R proteins (Figure 6D). Similar staining results were observed in the M1A and G10 cells (Figure S6A, Supporting Information). Notably, the IL‐11R signals in NK cells were far weaker compared to those in the cancer cells (Figure 6D). In agreement with the immunostaining findings, WB analysis demonstrated that NK cells expressed very low levels of IL‐11R compared to cancer cells (Figure 6E).

IL‐11 can affect target cells through either classical or trans‐signaling pathways.^[^ ^29^, ^30^, ^31^, ^32^ ^]^ In classical signaling, IL‐11 binds to IL‐11R on the surface of the target cell, activating downstream signaling pathways with the assistance of gp130. In trans‐signaling, IL‐11 secreted by effector cells forms complexes with soluble IL‐11R (sIL‐11R) that is shed from the effector cells. These IL‐11/sIL‐11R complexes then bind to gp130 on the surface of target cells, activating downstream pathways (Figure 6F). Our results showed that NK cells expressed low levels of IL‐11R but had sufficient gp130 (Figure 6D,E), suggesting that cancer cells might inhibit NK cell proliferation through IL‐11 trans‐signaling. To test this hypothesis, we should be able to detect sIL‐11R in the CM from cancer cells. Using ELISA, we detected the presence of sIL‐11R in the CM from cancer cells. Notably, there were no significant differences in sIL‐11R levels between the 231‐GFP and 231‐LC cells or the M1A and G10 cells (Figure 6G,H). We further confirmed the existence of IL‐11/sIL‐11R complexes in the CM from 231‐LC cells using co‐immunoprecipitation (Co‐IP) assays (Figure 6I). One thing that needs to be mentioned is that there was a slight shift of the major lower band of sIL‐11R and IL‐11 band in the WB after immunoprecipitation, which requires further study to explain. Immunostaining revealed that 15 min after adding the CM from 231‐LC cells to NK cells, there was an enrichment of IL‐11R signals on the surface of NK cells, indicating that sIL‐11R bound to the NK cell membrane (Figure 6J).

We then treated NK cells cultured in CM from 231‐LC cells with sgp130Fc, a specific inhibitor for IL‐11 trans‐signaling.^[^ ^33^, ^34^, ^35^ ^]^ The results indicated that sgp130Fc reversed the inhibitory effects of the CM on NK cell proliferation at commonly used concentrations of 50 and 100 ng mL^−1^ (Figure S6B,C, Supporting Information). For trans‐signaling, both IL‐11 and sIL‐11R are crucial for activating downstream pathways. We introduced anti‐IL‐11 neutralizing antibodies into the CM from 231‐LC cells. ELISA assays demonstrated that the addition of these antibodies led to a 95.3% reduction in the IL‐11 levels within the CM (Figure 6K). Importantly, after neutralizing IL‐11, the inhibitory effects of the CM from 231‐LC cells on NK cell proliferation were reversed (Figure 6L,M). Previous studies have indicated that membrane‐bound IL‐11R can be cleaved by the metalloproteinase ADAM10, resulting in the formation of sIL‐11R.^[^ ^32^, ^35^, ^36^, ^37^, ^38^, ^39^ ^]^ We first assessed the expression of ADAM10 in the M1A, G10, 231‐GFP, and 231‐LC cells using WB. The results showed that all these cancer cells expressed ADAM10 (Figure S6D, Supporting Information). Subsequently, we added a selective ADAM10 inhibitor, GI254023X (GI), to the culture media of the 231‐LC cells to decrease the levels of sIL‐11R in the CM.^[^ ^32^, ^37^ ^]^ The ELISA results indicated that GI was most effective at a concentration of 3 µM (Figure 6N). We then treated 231‐LC cells with GI at this concentration. Similar to IL‐11 neutralization, with sIL‐11R shedding inhibition, the CM from 231‐LC cells could no longer inhibit NK cell proliferation (Figure 6O,P).

**Figure 7:**
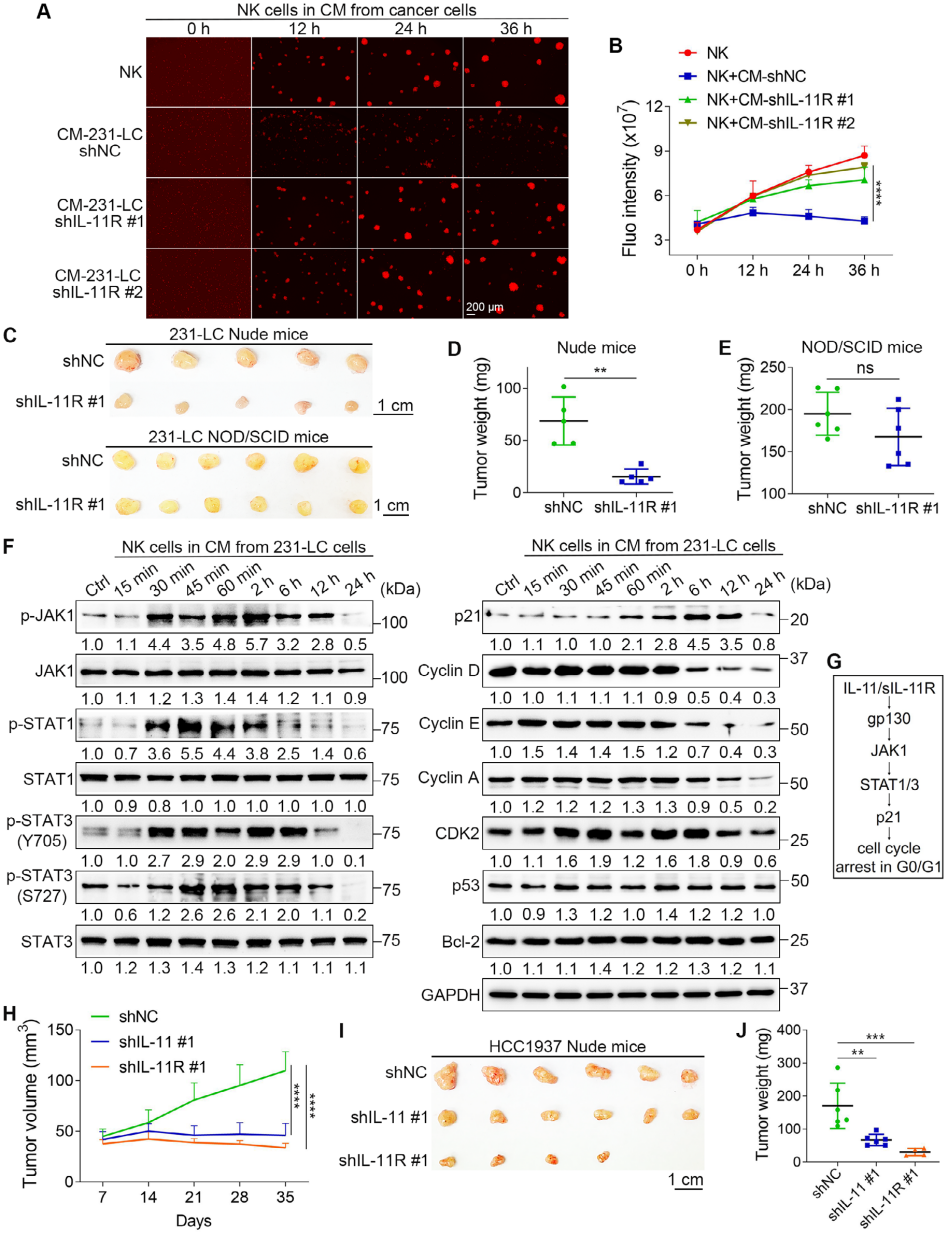
IL‐11 inhibits the proliferation of NK cells through the JAK1/STAT1/3 signaling pathway. A) Fluorescent images show the proliferation of NK cells cultured in CM from IL‐11R‐knockdown 231‐LC cells. B) Quantified results show the total fluorescence intensities of NK cells in (A) (*n* = 4). C–E) The tumor weights of control and IL‐11R‐knockdown 231‐LC cells in Nude (*n* = 5) and NOD/SCID mice (*n* = 6). F) WB analysis of the downstream pathways of IL‐11 trans‐signaling (*n* = 3). G) The proposed downstream signaling pathway of IL‐11 trans‐signaling. H) The tumor volumes of control and IL‐11‐ or IL‐11R‐knockdown HCC1937 cells (*n* = 6). I) The images of dissected tumors. J) Quantitative results show the tumor weights (*n* = 6). The sizes of scale bars are indicated in each image. The data were presented as mean ± SD. Statistical significance was determined by two‐way ANOVA (B, H), one‐way ANONA (J), or *t*‐test (D, E). ^**^ *p* < 0.01, ^***^ *p* < 0.001, ^****^ *p* < 0.0001, ns: not significant.

In addition to inhibiting sIL‐11R shedding, knocking down IL‐11R in cancer cells should also reverse the inhibitory effects on NK cell proliferation. Using shRNAs, we significantly reduced the expression of IL‐11R in 231‐LC cells, as confirmed by qPCR and WB (Figure S7A,B, Supporting Information). After IL‐11R knockdown, the 231‐LC cells became more sensitive to NK cell‐mediated killing and were no longer able to inhibit NK cell proliferation (Figure S7C,D, Supporting Information). Furthermore, the levels of IFNγ in the coculture media increased by 39.0% following IL‐11R knockdown (Figure S7E, Supporting Information). The CM from IL‐11R‐knockdown 231‐LC cells no longer inhibited the proliferation or DNA synthesis of NK cells (**Figure** **7**A,B; Figure S7F, Supporting Information). In orthotopic tumor models, tumor growth decreased by 77.4% in Nude mice after IL‐11R was knocked down in 231‐LC cells. In contrast, IL‐11R knockdown did not affect tumor growth in NOD/SCID mice (Figure 7C–E). These findings indicated that cancer cells resisted NK cell‐mediated killing and inhibited NK cell proliferation through IL‐11 trans‐signaling.

To investigate the downstream pathways of IL‐11 trans‐signaling, we cultured NK cells using CM from 231‐LC cells and collected NK cell lysates at various time points for WB analysis (Figure 7F). We focused on the JAK/STAT signaling pathway, which is a common downstream pathway activated by IL‐11.^[^ ^36^, ^40^, ^41^, ^42^, ^43^, ^44^, ^45^, ^46^, ^47^ ^]^ Our results demonstrated that the level of phosphorylated JAK1 (p‐JAK1) increased at 30 min post‐treatment and remained elevated for several hours. Then, p‐JAK1 levels gradually decreased over time, becoming barely detectable by 24 h. The total JAK1 levels showed minimal change during this period. We observed similar trends in the levels of p‐STAT1, p‐STAT3 (ser727), and p‐STAT3 (s705). p21 has been reported to be a key factor for cell cycle arrest, and it is directly regulated by STAT1 and STAT3.^[^ ^48^, ^49^, ^50^, ^51^, ^52^, ^53^ ^]^ The WB results indicated that p21 levels began to rise at 60 min and sustained this increase for at least 12 h. In our study, we also found that NK cell proliferation was blocked at the G0/G1 phase of the cell cycle following CM treatment. Consequently, we measured the levels of cyclin D, cyclin E, cyclin A, and CDK2. The results showed that the levels of cyclin D, cyclin E, and cyclin A began to decline at 6 h and were undetectable by 24 h. CDK2 levels decreased slightly after 24 h of CM treatment. We also assessed levels of the apoptotic regulators p53 and Bcl‐2; their levels remained unchanged, which aligned with our findings that NK cells did not undergo apoptosis with CM treatment (Figure S4F,G, Supporting Information). Based on these data, the downstream pathway of IL‐11 trans‐signaling was provided (Figure 7G).

So far, we have demonstrated that G10 cells and 231‐LC cells can resist NK cell‐mediated killing and inhibit the proliferation of NK cells through IL‐11 trans‐signaling. It is important to note that both G10 and 231‐LC cells are derived from MDA‐MB‐231 cells. To further validate our findings in additional TNBC cell lines, we assessed the expression levels of IL‐11 and IL‐11R in three more TNBC cell lines, in addition to MDA‐MB‐231 cells. The data revealed that all tested TNBC cell lines expressed IL‐11, with HCC1937 cells exhibiting the highest expression levels. Interestingly, the levels of IL‐11R were similar across these four TNBC cell lines (Figure S8A, Supporting Information). Next, we reduced the expression levels of IL‐11 (Figure S8B,C, Supporting Information) or IL‐11R (Figure S8D,E, Supporting Information) in HCC1937 cells using shRNAs. The results from coculture experiments indicated that HCC1937 cells became sensitive to NK cell‐mediated killing, and they could no longer inhibit NK cell proliferation after knocking down either IL‐11 or IL‐11R (Figure S8F,G, Supporting Information). The levels of IFNγ in the coculture media increased significantly after the knockdown of IL‐11 or IL‐11R (Figure S8H, Supporting Information). In addition, CM from IL‐11‐ or IL‐11R‐knockdown HCC1937 cells did not inhibit the proliferation or DNA synthesis of NK cells (Figure S8I–K, Supporting Information). More importantly, the knockdown of IL‐11 or IL‐11R in HCC1937 cells resulted in a significant reduction in tumor growth in Nude mice, by 60.7% and 82.1% respectively (Figure 7H–J).

### 2.6 IL‐11 Is Highly Expressed in Tumor Tissues from Mice and TNBC Patients, and Its Expression Is Negatively Correlated with the Number of NK Cells in the TME

We first measured the number of NK cells in 231‐LC orthotopic tumors from Nude mice using immunohistochemical (IHC) staining. The results indicated that after IL‐11 knockdown, the number of NK cells in the TME, as identified by the DX5 marker, increased 3.4‐fold. Interestingly, the level of IL‐11R in the orthotopic tumors remained unchanged following IL‐11 knockdown (Figure S9A–D, Supporting Information). Additionally, we stained orthotopic tumors from NOD/SCID mice and could barely detect DX5‐positive cells (Figure S9E, Supporting Information), confirming the deficiency of NK cells in NOD/SCID mice.

**Figure 8:**
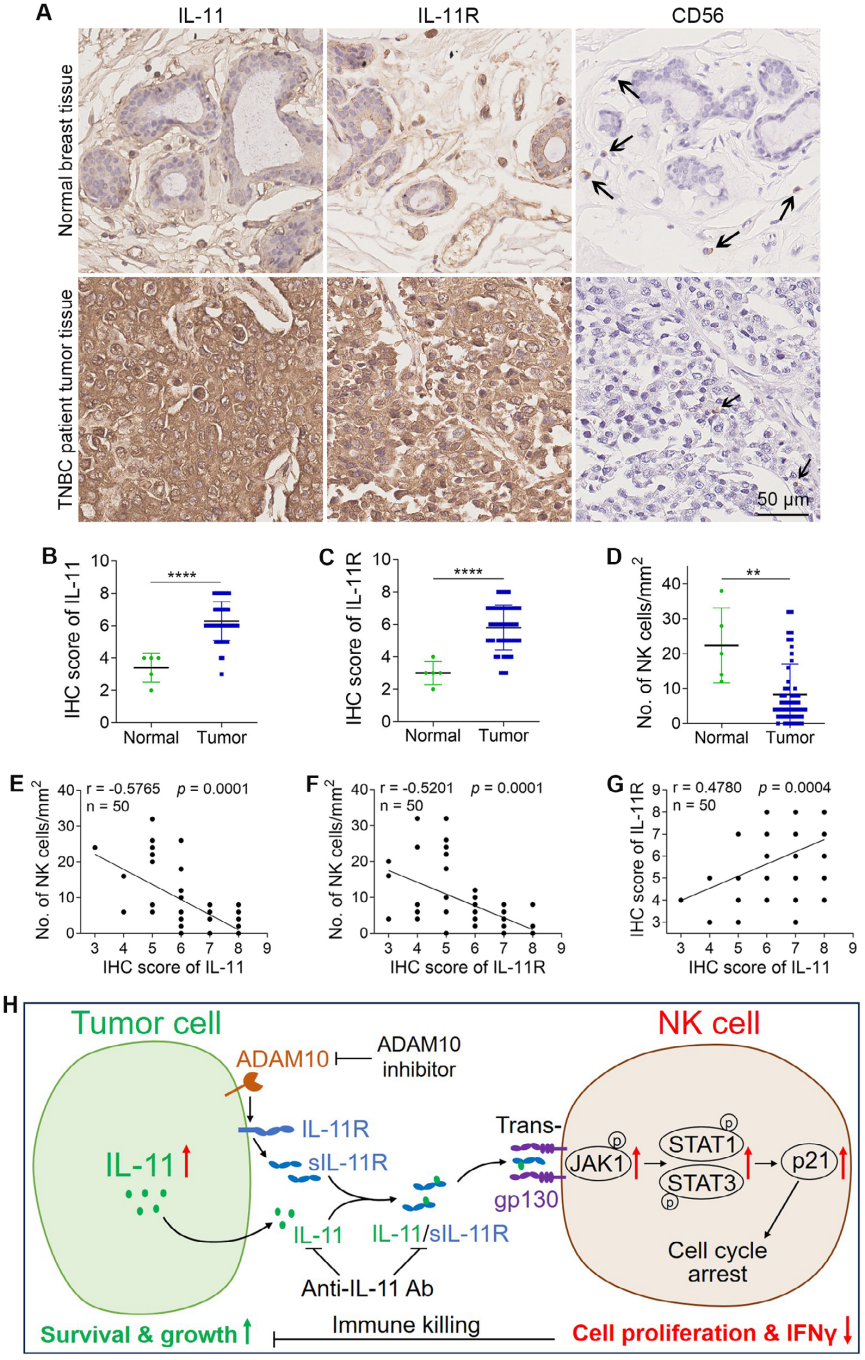
IL‐11 is highly expressed in tumor tissues, and its expression is negatively correlated with the number of NK cells in the TME. A) IHC images show the staining of IL‐11, IL‐11R, and CD56 in tumor tissues from TNBC patients. The NK cells are indicated with arrows. B,C) IHC scores of IL‐11 and IL‐11R (*n* = 5 for normal tissues and *n* = 50 for tumor tissues). D) Number of NK cells in the TME (*n* = 5 for normal tissues and *n* = 50 for tumor tissues). E) The correlation between IL‐11 IHC score and NK cell number (*n* = 50). F) The correlation between IL‐11R IHC score and NK cell number (*n* = 50). G) The correlation between IL‐11 IHC score and IL‐11R IHC score (*n* = 50). H) A diagram illustrates how TNBC cells resist NK‐mediated killing through IL‐11. IL‐11 secreted by TNBC cells forms complexes with sIL‐11R. These IL‐11/sIL‐11R complexes then bind to the gp130 receptor on NK cells, activating the downstream signaling pathway and inhibiting the proliferation and IFNγ production of NK cells. Consequently, TNBC cells survive better and grow into larger tumors. The data were presented as mean ± SD. Statistical significance was determined by *t*‐test (B, C, D). ^**^ *p* < 0.01, ^****^ *p* < 0.0001.

Then, we measured the levels of IL‐11, IL‐11R, and the human NK cell marker CD56 in tumor tissues from TNBC patients. Compared to normal breast tissues, the levels of IL‐11 and IL‐11R in the TME were significantly higher, while the number of NK cells decreased by 63.0% (**Figure** **8**A–D). More importantly, we found a negative correlation between the number of NK cells in the TME and the levels of IL‐11 or IL‐11R (Figure 8E,F). Interestingly, the expression levels of IL‐11 and IL‐11R were positively correlated with each other (Figure 8G). These data indicated that under clinical conditions, TNBC cells reduced the number of NK cells in the TME by upregulating IL‐11.

Collectively, we propose that TNBC cells secrete IL‐11, which forms complexes with sIL‐11R. These IL‐11/sIL‐11R complexes then bind to the gp130 receptor on NK cells. This binding activates the downstream JAK1/STAT1/3/p21 signaling pathway, which blocks the cell cycle of NK cells. As a result, the proliferation and IFNγ production of NK cells are reduced, leading to a decreased killing ability. Consequently, TNBC cells are better able to survive and grow into larger tumors (Figure 8H).

## 3 Discussion

NK cells are a critical part of the innate immune system, essential for controlling tumor progression. However, in the TME, NK cells often become exhausted, which weakens their ability to eliminate cancer cells. While many studies have explored the mechanisms of NK cell exhaustion, there is still limited research specifically focused on TNBC.^[^ ^6^, ^7^, ^54^, ^55^ ^]^ Our study found that TNBC cells resist NK cell‐mediated killing by inhibiting NK cell proliferation and IFNγ secretion through IL‐11 trans‐signaling. This discovery significantly enhances our understanding of NK cell exhaustion in TNBC.

IL‐11 has diverse biological functions, including roles in hematopoiesis, immune regulation, tissue repair, and aging.^[^ ^46^, ^56^, ^57^ ^]^ Recently, its involvement in cancer progression has drawn increasing attention. Researchers have found that IL‐11 is significantly overexpressed in various cancer types, including TNBC, and patients with elevated IL‐11 levels often experience poorer prognoses.^[^ ^45^, ^58^, ^59^, ^60^, ^61^, ^62^ ^]^ In TNBC, intra‐tumoral heterogeneity is a notable characteristic. Studies indicate that subclones with high IL‐11 expression tend to develop larger tumors compared to those with overexpression of other genes.^[^ ^63^ ^]^ Bones are a common site for distant metastasis in breast cancer. Research examining specific subpopulations of TNBC cells identified a group with heightened propensity for bone metastasis, leading to the validation of overexpressed genes. Among these, IL‐11 emerged as a crucial factor promoting bone metastasis in TNBC. Further investigations revealed that IL‐11 can enhance osteolysis, thereby facilitating the bone metastasis process.^[^ ^64^, ^65^ ^]^ Several microRNAs have been identified as suppressors of IL‐11‐driven bone metastasis in TNBC.^[^ ^66^, ^67^ ^]^ Chemotherapy resistance remains a significant challenge in treating TNBC. Studies have shown that IL‐11 secreted by TNBC cells plays a critical role in this resistance. Inhibiting IL‐11 expression has been found to restore the sensitivity of TNBC cells to chemotherapy drugs such as doxorubicin and paclitaxel.^[^ ^68^ ^]^

Our study revealed a novel role for IL‐11 in promoting the progression of TNBC. We discovered that IL‐11, which is secreted by TNBC cells, can inhibit the proliferation and IFNγ production of NK cells by activating the JAK1/STAT1/3 signaling pathway. Interestingly, previous research has demonstrated that IL‐11 can induce senescence and reduce the number of uterine NK cells during pregnancy, also through the activation of STAT3.^[^ ^69^ ^]^ In this study, we utilized NK‐92MI cells, which are frequently used to investigate NK cell function and serve as a source for clinical CAR‐NK cell production.^[^ ^70^ ^]^ Future research should examine whether the proliferation of peripheral blood mononuclear cells or spleen‐derived NK cells can also be inhibited by IL‐11 secreted by TNBC cells. Furthermore, it is important to investigate whether IL‐11 released from TNBC cells affects the normal developmental processes of NK cells.

In addition to its inhibitory effects on NK cells, some studies indicated that IL‐11 can also suppress T cell function. Specifically, IL‐11 has been found to inhibit the production of IL‐12 and IFNγ by T cells; however, it does not affect T cell proliferation.^[^ ^71^, ^72^ ^]^ A mouse model of colon cancer demonstrated that IL‐11 signaling promoted tumor growth, and the mechanism study revealed that IL‐11 achieved this effect by inhibiting CD4^+^ T cell‐mediated tumor‐killing responses.^[^ ^73^ ^]^ Furthermore, IL‐11 can indirectly inhibit T cell activity by promoting the differentiation of myeloid‐derived suppressor cells.^[^ ^74^ ^]^ These findings suggest that IL‐11 can negatively regulate the killing activity of immune cells, a function that cancer cells exploit to evade the immune response.

NK cell‐based therapy has advanced significantly in recent years. However, clinical trials have shown that the response rates for treating solid tumors, including TNBC, have been unsatisfactory. Blocking IL‐11 trans‐signaling has the potential to promote the proliferation of NK cells and enhance their killing ability against cancer cells. In the future, combining antibodies or inhibitors that block IL‐11 trans‐signaling with CAR‐NK therapy may improve therapeutic outcomes for TNBC.

## 4 Experimental Section

### Cell Lines and Cell Culture

TNBC cell lines M1A, G10, 231‐GFP, 231‐LC, MDA‐MB‐231, MDA‐MB‐468, BT‐549, and HCC1937 were cultured in Dulbecco's modified Eagle's medium (DMEM) (12100‐046, Thermo Fisher Scientific) supplemented with 10% fetal bovine serum (10270‐106, Gibco) and 1% penicillin‐streptomycin (15140‐122, Thermo Fisher Scientific). NK‐92MI and NK‐92MI‐tdT cells were cultured in α‐MEM (12000‐022, Thermo Fisher Scientific) supplemented with 0.02 mM folic acid (F8758‐5G, Sigma‐Aldrich), 0.2 mM inositol (I7508‐50G, Sigma‐Aldrich), 0.1 mM 2‐mercaptoethanol (M3148‐250ML, Sigma‐Aldrich), 12.5% horse serum (16050‐122, Thermo Fisher Scientific), 12.5% fetal bovine serum and 1% penicillin‐streptomycin.

### 2D and 3D Coculture

In the 2D coculture condition, 40 000 cancer cells were seeded into each well of a 48‐well plate. After the attachment of the cancer cells, 40 000 NK cells were added to each well. Fluorescent images were acquired at various time points during the coculture process using a Carl Zeiss LSM 880 confocal laser scanning microscope. The apoptotic rates of cancer cells were assessed through FRET imaging. In the 3D coculture condition, 2000 cancer cells were seeded into each well of a low attachment 96‐well plate. Then, 400 to 2000 NK cells were added to each well. Fluorescence images were obtained at multiple time points during the coculture process using a Carl Zeiss Axio Observer microscope.

### Calcein Release Assay

A total of 10 000 cancer cells were seeded into each well of a 96‐well plate. After the attachment of cancer cells, they were stained with calcein‐AM dye (C3099, Thermo Fisher Scientific) at a concentration of 2.5 µM for 30 min at 37 °C. Following the staining procedure, the cancer cells were washed twice with DMEM. Subsequently, 10 000 NK cells were added to each well in a total volume of 200 µL. After 6 h of coculture, the plate was centrifuged, and 100 µL of the supernatant from each well was collected to measure calcein release. The fluorescent signals were detected using a PerkinElmer Victor3 plate reader.

### Treating NK Cells with Conditioned Media (CM) from Cancer Cells

Cancer cells were cultured for 24 h, and the CM was collected through centrifugation. The effects of CM on the growth of NK cells were determined. A total of 2000 NK cells were cultured in different CM for various time points. Then, fluorescent images were captured using a Carl Zeiss Axio Observer microscope to assess the growth of NK cells. Additionally, the total fluorescence intensities of the NK cells were measured using ImageJ software.

### Cell Cycle Analysis

NK cells were cultured for 24 h with CM from various cancer cells. After that, the NK cells were collected and resuspended in 1 mL of phosphate‐buffered saline (PBS). Subsequently, 3 mL of precooled absolute ethanol was added to this NK cell suspension. The NK cells were fixed at 4 °C. Following fixation, propidium iodide (PI) staining was performed according to the instructions provided in the cell cycle analysis kit (MA0334, Meilunbio). The cell cycle was then analyzed using a CytoFLEX flow cytometer.

### Annexin V Staining

After treating the NK cells for 24 h with CM from different cancer cells, the NK cells were collected and washed twice with cold PBS. Following the washes, 100 µL of binding buffer was added to each sample. Next, 5 µL of FITC Annexin V (A13201, Invitrogen) and 10 µL of PI were added, and the samples were incubated in the dark for 15 min. After incubation, 400 µL of Annexin V binding buffer was added to resuspend the cells. The apoptotic rates were then analyzed using a CytoFLEX flow cytometer.

### EdU Incorporation Assay

NK cells were cultured in different CM for 22 h. Subsequently, EdU solution (C0071S, Beyotime) was added to the NK cells at a final concentration of 20 µM, and the cells were cultured for an additional 2 h. The NK cells were then collected and fixed with 4% paraformaldehyde (PFA) (30525‐89‐4, Sigma‐Aldrich) for 15 min, followed by three washes with PBS. Next, 1 mL of permeabilization buffer (P0097, Beyotime) was added. Permeabilization was performed overnight at 4 °C. Following three washes with PBS, 0.5 mL of fresh click additive solution was added, and the cells were incubated in the dark at room temperature for 30 min. After additional washing with PBS, Hoechst 33 342 (H‐3570, Thermo Fisher Scientific) was used to stain nuclei at a dilution of 1:1000 for 10 min. Finally, after three washes, the EdU‐positive cells were captured using a Carl Zeiss Axio Observer microscope.

### Granule Secretion Analysis

Cancer cells and NK cells were cocultured at a ratio of 1:1 for 20 h. Then, monensin (M5273, Sigma‐Aldrich) was added to the culture at a final concentration of 2 µM to block granule secretion. After an additional 4 h of coculture, the NK cells were collected and resuspended in 5 mL of wash buffer. These NK cells were then fixed with 4% PFA for 20 min. After fixation, the NK cells were washed and permeabilized for 10 min. Subsequently, 5 µL of allophycocyanin‐conjugated IgG control, as well as anti‐PRF1 and anti‐GZMB primary antibodies, were added to the NK cells. The cells were incubated for 1 h at 4 °C in the dark. Finally, the NK cells were washed twice and analyzed using a BD Accuri C6 cell sorter.

### Enzyme‐Linked Immunosorbent Assay (ELISA)

A total of 40 000 cancer cells and 40 000 NK cells were cocultured in each well of a 48‐well plate for 24 h. Then, the cell culture supernatants were collected to assess the release of IFNγ from the NK cells using an IFNγ ELISA kit (430 104, Biolegend). Similarly, 40 000 cancer cells were cultured in each well of a 48‐well plate for 24 h, and the supernatants were collected to assess the levels of IL‐11 and sIL‐11R using a human IL‐11 ELISA kit (D1100, R&D Systems) and a human IL‐11RA ELISA kit (LS‐F8919‐1, LSBio Biotech Company). A standard curve was generated based on the concentrations of the standard proteins and their corresponding OD values. Subsequently, the concentration of each sample was calculated using this standard curve.

### Cell Transfection and Lentiviral Production

HEK 293T cells were seeded into each well of a 6‐well plate containing complete DMEM. After cell attachment, the culture medium was replaced with serum‐free DMEM. A core plasmid, along with two helper plasmids (VSV‐G and dR8.2), was transfected into HEK 293T cells. After 6 h of transfection, the serum‐free medium was replaced with complete DMEM. Viral particles were harvested at 36 and 72 h post‐transfection. These viral particles were then used to infect target cells for 24 to 36 h. Positive target cells were selected based on resistance to blasticidin (ant‐bl‐1, InvivoGen). All knockdown and overexpression plasmids were ordered from VectorBuilder. The transcript ID for the IL‐11 overexpression plasmid is NM_000641.4, and the length of the coding DNA sequence is 600 bp. The detailed shRNA sequences are provided in Table S1 (Supporting Information).

### RNA Sequencing

Cancer cells were collected and lysed with 1 mL of TRIzol solution (15 596 018, Thermo Fisher Scientific). These samples were then sent to Novogene Company (Tianjin, China) for RNA sequencing and data analysis. Differentially expressed genes were identified based on read count values, with a threshold of a log2 fold change >1.

### Quantitative Polymerase Chain Reaction (qPCR)

Cells were lysed with 600 µL of TRIzol solution. RNAs were extracted and then reverse‐transcribed with a cDNA synthesis kit (1 708 891, Bio‐Rad). Finally, the cDNAs were amplified with specific primers and SYBR Green Supermix (172‐5124, Bio‐Rad) on a CFX96 Real‐time PCR Detection System. GAPDH served as the internal control. Results were analyzed using the 2‐∆∆Ct method. The primers utilized in this study are listed in Table S2 (Supporting Information).

### Western Blotting (WB)

Cells were lysed in radioimmunoprecipitation assay buffer containing protease inhibitors. Afterward, the cell lysates were centrifuged, and the supernatants were collected. The protein concentrations were measured using a Bio‐Rad Protein Assay Dye Reagent (5 000 006, Bio‐Rad). Next, the proteins were separated using sodium dodecyl sulfate‐polyacrylamide gel electrophoresis. After electrophoresis, the proteins were transferred to nitrocellulose membranes (1 620 112, Bio‐Rad). The membranes were then blocked with 5% non‐fat dry milk at room temperature for 1 h. Following this, the membranes were incubated with various primary antibodies at 4 °C overnight. Afterward, the membranes were washed three times and subsequently incubated with corresponding secondary antibodies at room temperature for 1 h. Finally, the membranes were washed three times and developed using Western ECL substrate (1 705 061, Bio‐Rad). The antibodies used in this study are listed in Table S3 (Supporting Information).

### Immunofluorescence Staining

Cultured cells were washed with PBS and then fixed with 4% PFA at room temperature for 15 min. After fixation, the cells were washed three times with PBS and then permeabilized with 0.2% Triton X‐100 at room temperature for 15 min. Following permeabilization, the cells were blocked with 3% BSA/PBS at room temperature for 1 h. After blocking, the cells were incubated overnight at 4 °C with diluted primary antibodies. Next, the cells were washed three times with PBS and incubated for 1 h at room temperature in the dark with diluted fluorescent dye‐conjugated secondary antibodies. Subsequently, the cells were washed three times with PBS and incubated with Hoechst 33 342 (1:1000 dilution) at room temperature for 20 min in the dark. Finally, fluorescent images were captured using a Carl Zeiss LSM 880 confocal laser scanning microscope. The antibodies are listed in Table S3 (Supporting Information).

### Co‐Immunoprecipitation Assay

A total of 231‐LC cells were seeded in a 10 cm culture dish with complete DMEM. After cell attachment, the cells were washed twice with PBS. Then, the cells were cultured in serum‐free DMEM for 24 h. After that, the CM was collected and filtered using a 0.22 µm filter. The CM was then concentrated using a 10 kDa ultra centrifugal filter (UFC9010, Millipore) at 5000 g and at 4 °C. 400 µL of concentrated CM, 600 µL of IP buffer, and 10 µL of IL‐11 or IgG isotype control antibody were incubated in a 1.5 mL tube with gentle rotation at 4 °C for 12 h. Following this, 50 µL of protein A/G agarose beads were added to the tube for an additional 2 h of incubation at room temperature. After that, the mixture was centrifuged and washed three times with IP buffer. Then, 75 µL of elution buffer was added. The supernatant containing the protein and antibody complex was collected and applied for subsequent WB analysis. The antibodies are listed in Table S3 (Supporting Information).

### IL‐11 Neutralization

A total of 40 000 231‐LC cells were seeded into each well of a 48‐well plate. After cell attachment, an anti‐IL‐11 antibody (XP‐5164, ProSci Incorporated) was added at a final concentration of 10 µg mL^−1^. The cells were then cultured for an additional 12 h, after which the CM was collected. This CM was used for the ELISA analysis and to treat NK cells.

### Immunohistochemistry Staining

The experimental procedures adhered to the guidelines provided in the immunohistochemistry kit manual (ab64264, Abcam). In brief, mouse tumor slides and clinical TNBC breast cancer tissue chips (Guangzhou Wozhe Biotech Company) were first deparaffinized and rehydrated. Following this, the slides were incubated in sodium citrate antigen retrieval buffer at 95–100 °C for 10 min. After antigen retrieval, the slides were incubated with diluted primary antibodies at 4 °C for 12 h. Subsequently, the slides were washed three times and incubated with biotin‐conjugated secondary antibodies at room temperature for 1 h. The slides were then developed according to the kit's instructions. Finally, the slides were stained with hematoxylin and mounted for scanning. The IHC scores were assessed using the Allred scoring method and quantified in a blinded manner. The antibodies are listed in Table S3 (Supporting Information).

### Orthotopic Tumor Models

Cancer cells (2 × 10^6^) were injected into the fat pads of Nude or NOD/SCID mice. After injection, tumor volumes were measured throughout the experiments. At the end of the experiments, the tumor‐bearing mice were sacrificed, and the tumors were dissected for further analysis. All animal experiments were approved by the Animal Ethics Committee of the University of Macau (UMARE‐025‐2017 and UMARE‐026‐2017).

### Statistics

All data are presented as the mean ± standard deviation. Statistical significance was evaluated by one‐way or two‐way analysis of variance (ANOVA) or *t*‐test using GraphPad Prism 9.0 software. Significance levels are indicated as follows: ^*^ *p* < 0.05, ^**^ *p* < 0.01, ^***^ *p* < 0.001, ^****^ *p* < 0.0001, ns: not significant.

## Conflict of Interest

The authors declare no conflict of interest.

## Author Contributions

H.Y., H.J., and K.Q.L. designed the experiments. H.Y., H.T., and H.J. performed the experiments. H.Y., H.J., R.W., L.C., and K.Q.L. analyzed the data. H.Y. and H.J. prepared the manuscript. K.Q.L. revised the manuscript. K.Q.L. supervised this study.

## Supporting information



Supporting Information

## Data Availability

The data that support the findings of this study are available from the corresponding author upon reasonable request.
