# Supplementary material for: Triple‐Negative Breast Cancer Cells Resist Natural Killer Cell‐Mediated Killing Through Interleukin‐11 Trans‐Signaling
Source: Adv Sci (Weinh). 2025 Nov 3;13(4):e15772. doi: 10.1002/advs.202515772 (PMC12822480; doi:10.1002/advs.202515772)
Supplement: Supplementary file 1 — Supporting Information [file ADVS-13-e15772-s001.docx]

**Triple-Negative Breast Cancer Cells Resist Natural Killer Cell-Mediated Killing through Interleukin-11 Trans-Signaling**

Hongmei Yang^1^, Hao Jia^1^, Renfei Wu^1^, Haibo Tong^1^, Liping Chen^1^, Kathy Qian Luo^1,2^*

^1^ Department of Biomedical Sciences, Faculty of Health Sciences, University of Macau, Taipa, Macao SAR, China

^2^ Ministry of Education Frontiers Science Center for Precision Oncology, University of Macau, Taipa, Macao SAR, China

*For correspondence: [kluo@um.edu.mo](mailto:kluo@um.edu.mo)

**
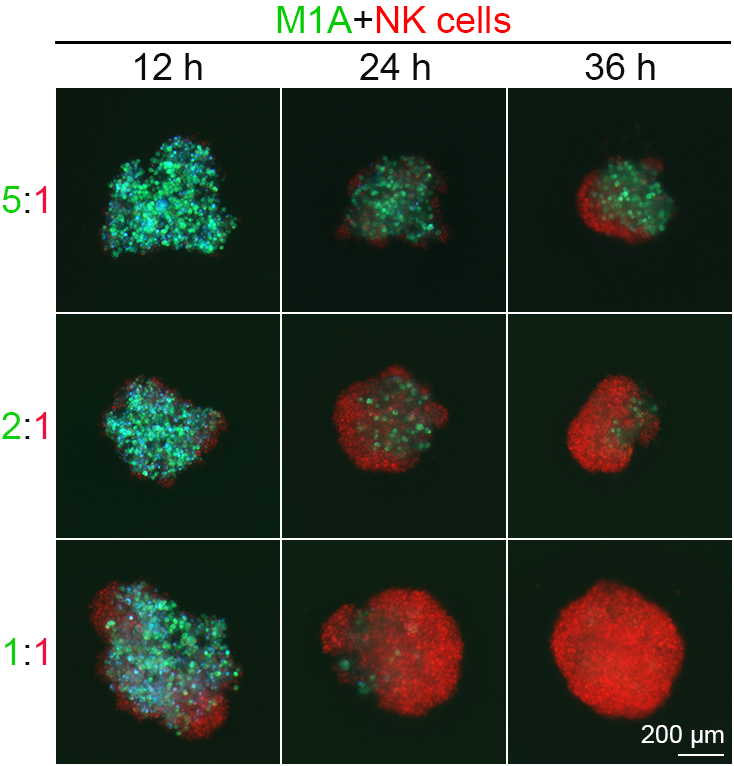
**

**Figure S1.** A proportion of M1A cells resist NK cell-mediated killing. Fluorescent images show the coculture of M1A with NK-92MI-tdT cells at different ratios under 3D conditions.

**
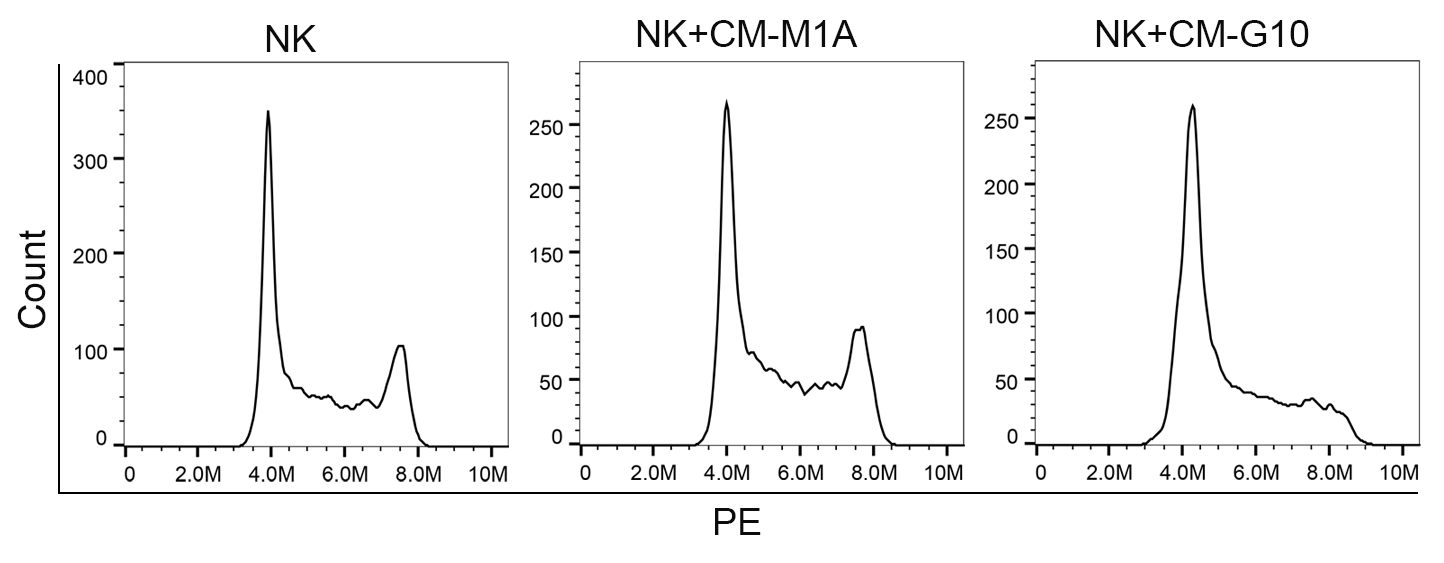
**

**Figure S2.** The cell cycle of NK cells is blocked when cultured in the CM from G10 cells. The cell cycle distribution of NK cells after being cultured for 24 h in different media.

**
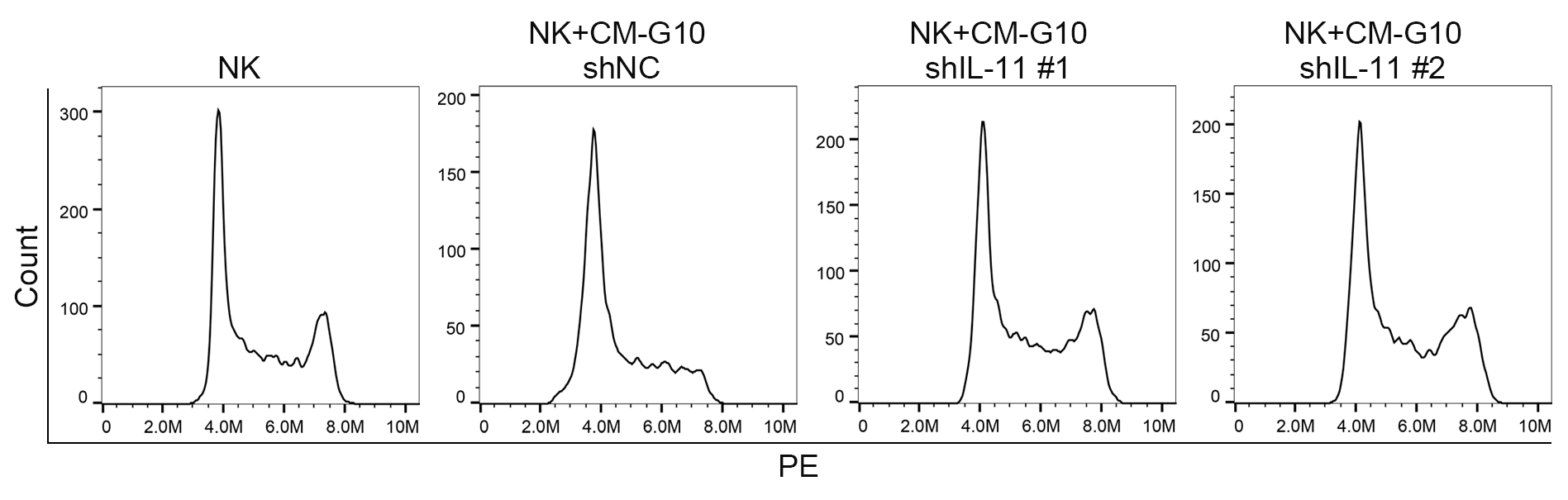
**

**Figure S3.** The cell cycle block of NK cells is reversed when cultured in the CM from IL-11-knockdown G10 cells. The cell cycle distribution of NK cells after being cultured for 24 h in different media.


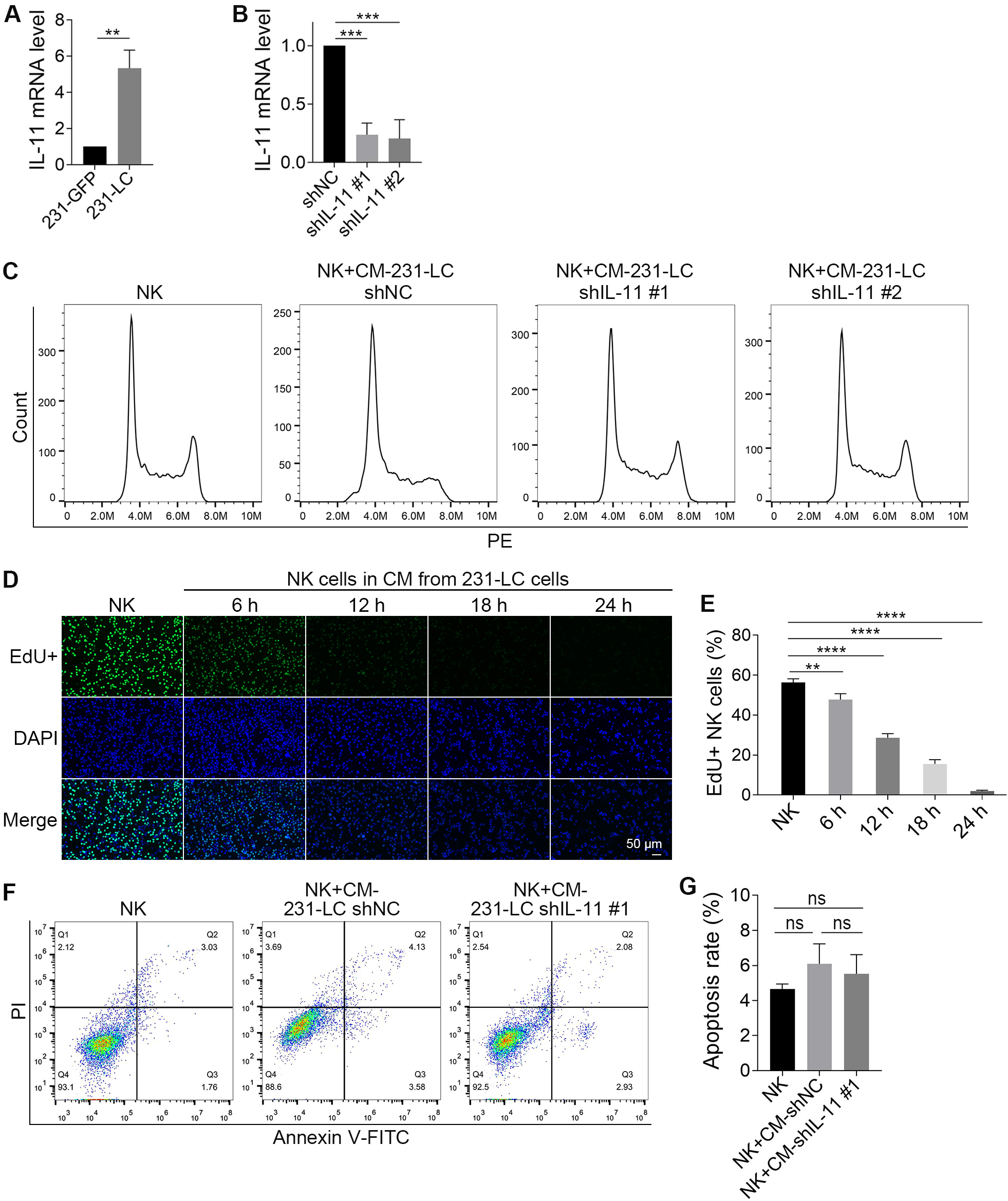


**Figure S4.** The cell cycle block and DNA synthesis inhibition of NK cells by the CM from 231-LC cells. A) The mRNA levels of IL-11 in 231-GFP and 231-LC cells were detected by qPCR (n=3). B) The knockdown of IL-11 in 231-LC cells was confirmed by qPCR (n=3). C) The cell cycle distribution of NK cells after being cultured for 24 h in CM from shNC and IL-11-knockdown 231-LC cells. D) Fluorescent images show the EdU-positive NK cells cultured in normal NK cell culture medium or CM from 231-LC cells. The size of the scale bar is indicated in the image. E) Quantified results show the percentages of EdU-positive NK cells (n=3). F, G) Flow cytometry results show the apoptotic rates of NK cells cultured for 24 h in normal NK cell culture medium, CM from shNC 231-LC cells, or CM from IL-11-knockdown 231-LC cells (n=3). The data were presented as mean ± SD. Statistical significance was determined by *t*-test (A) or one-way ANONA (B, E, G). ***p* < 0.01, ****p* < 0.001, *****p* < 0.0001, ns: not significant.


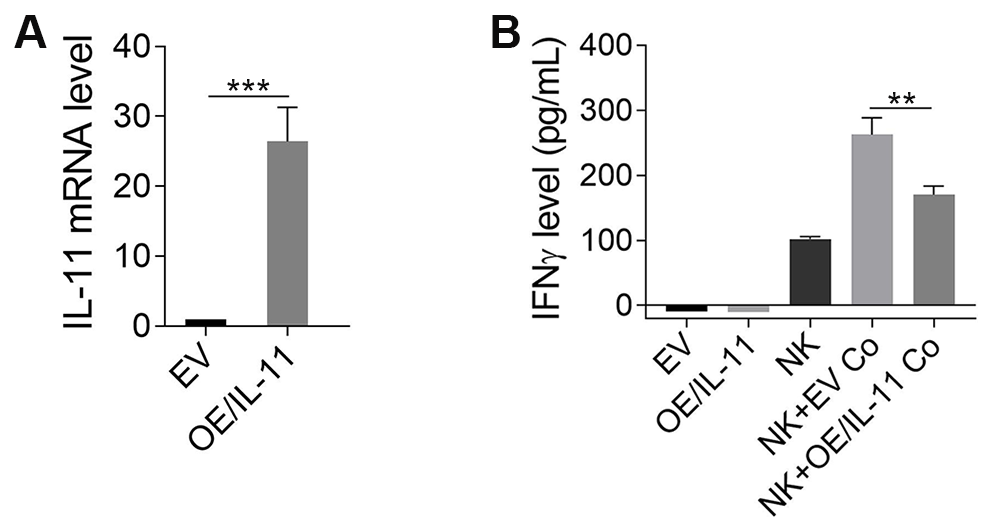


**Figure S5.** IL-11 overexpressing 231-GFP cells inhibit the IFNγ production of NK cells. A) The mRNA level of IL-11 was upregulated in IL-11 overexpressing 231-GFP cells (n=3). B) The concentrations of IFNγ in the monoculture or coculture media after 24 h of culture (n=4). The data were presented as mean ± SD. Statistical significance was determined by *t*-test (A) or one-way ANONA (B). ***p* < 0.01, ****p* < 0.001.

**
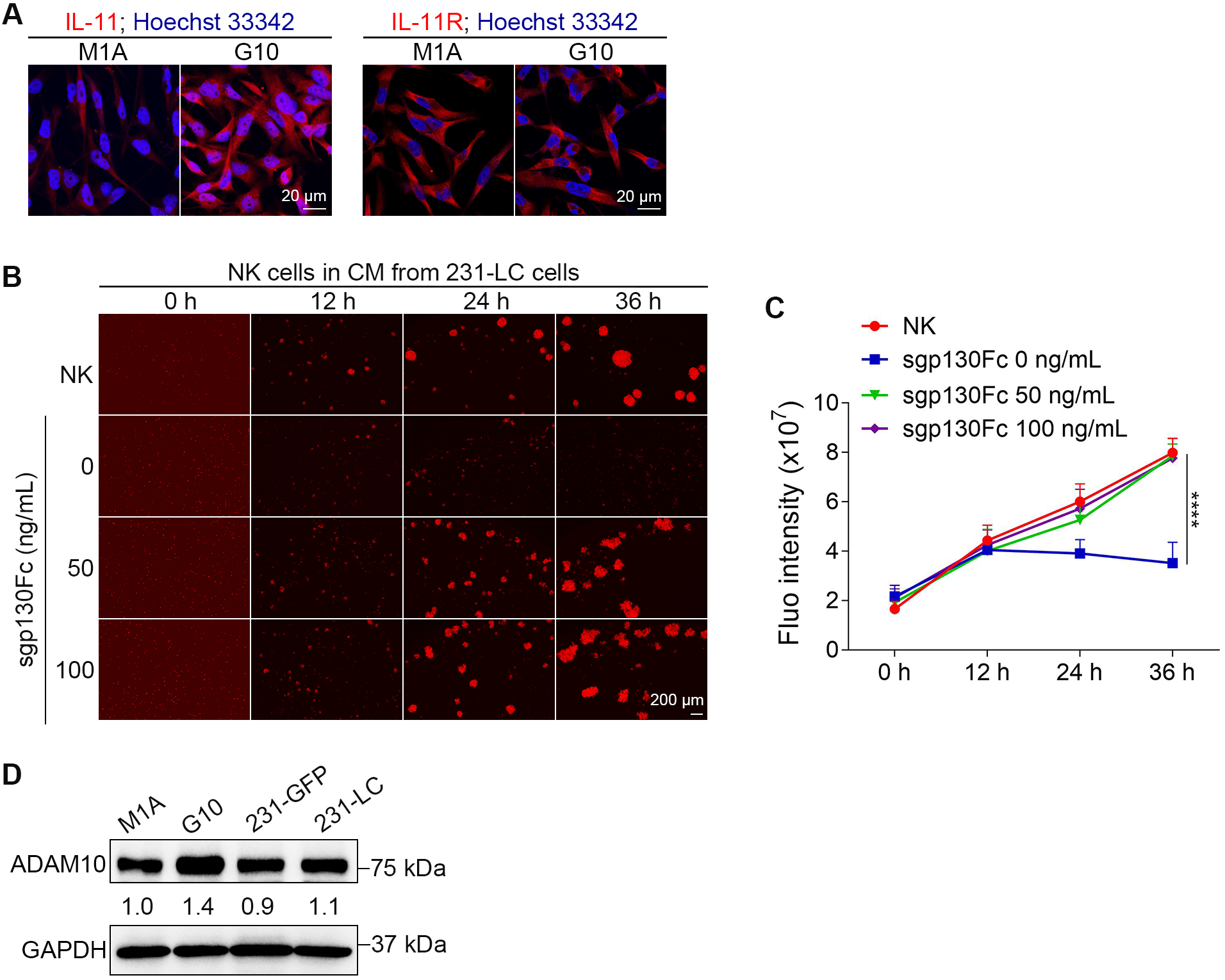
**

**Figure S6.** The expression levels of IL-11 and IL-11R in M1A and G10 cells. A) Immunostaining shows the levels of IL-11 and IL-11R in M1A and G10 cells. B) Fluorescent images show the proliferation of NK cells cultured in the CM from 231-LC cells with or without sgp130Fc at the concentrations of 50 and 100 ng/mL. C) Quantified results show the total fluorescence intensities of NK cells in (B) (n=4). D) WB shows the protein levels of ADAM10 in cancer cells (n=3). The sizes of scale bars are indicated in each image. The data were presented as mean ± SD. Statistical significance was determined by two-way ANOVA (C). *****p* < 0.0001.


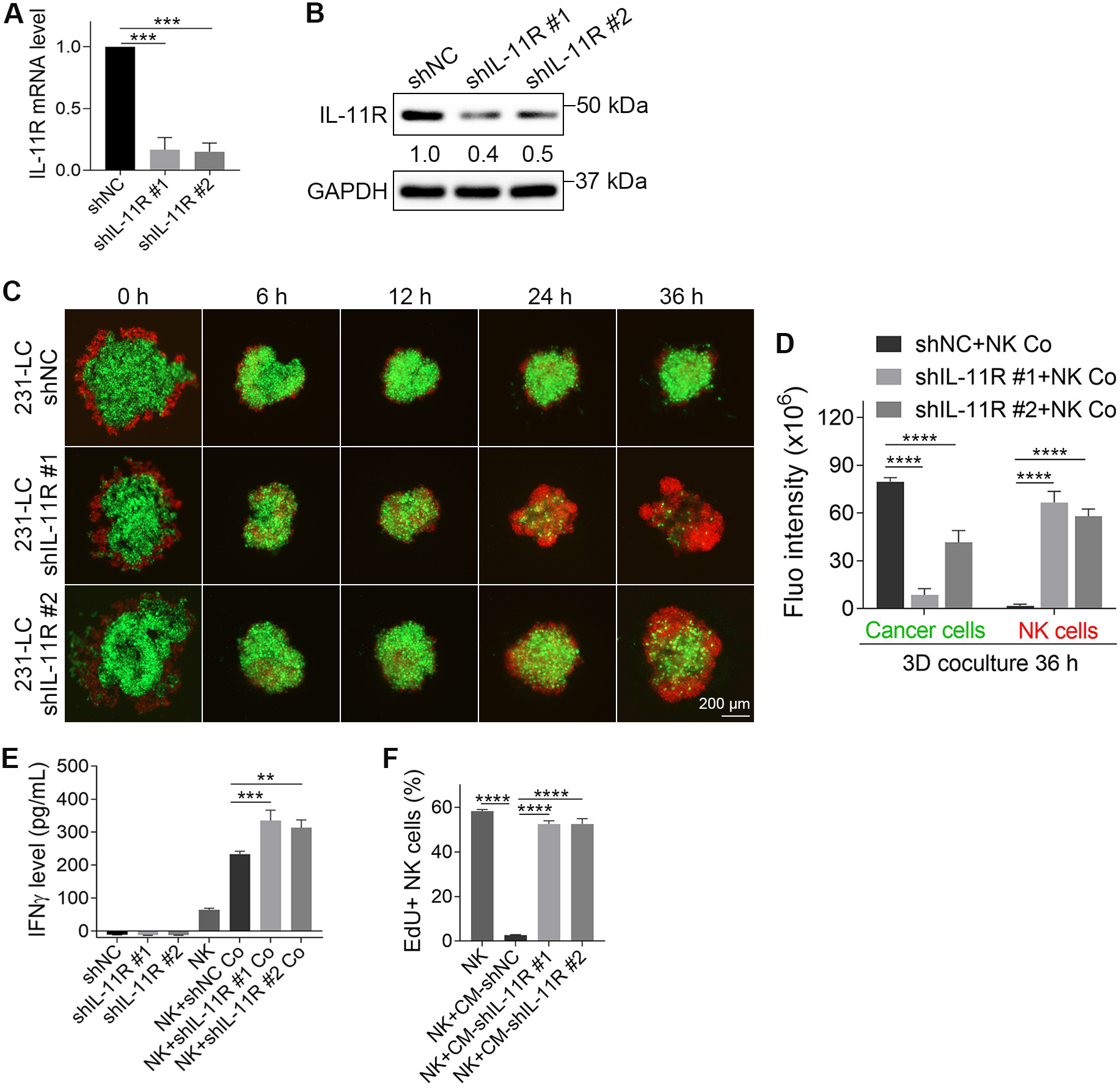


**Figure S7.** IL-11R-knockdown 231-LC cells are sensitive to NK cells. A, B) qPCR and WB show the knockdown of IL-11R in 231-LC cells (n=3). C) Fluorescent images show the coculture of IL-11R-knockdown 231-LC cells with NK cells. D) Quantified results show the total green and red fluorescence intensities after 36 h of coculture in (C) (n=3). E) The concentrations of IFNγ after 24 h of culture (n=3). F) The percentages of EdU-positive NK cells after culturing for 24 h in different media (n=3). The data were presented as mean ± SD. Statistical significance was determined by two-way ANOVA (D) or one-way ANONA (A, E, F). ***p* < 0.01, ****p* < 0.001, *****p* < 0.0001.


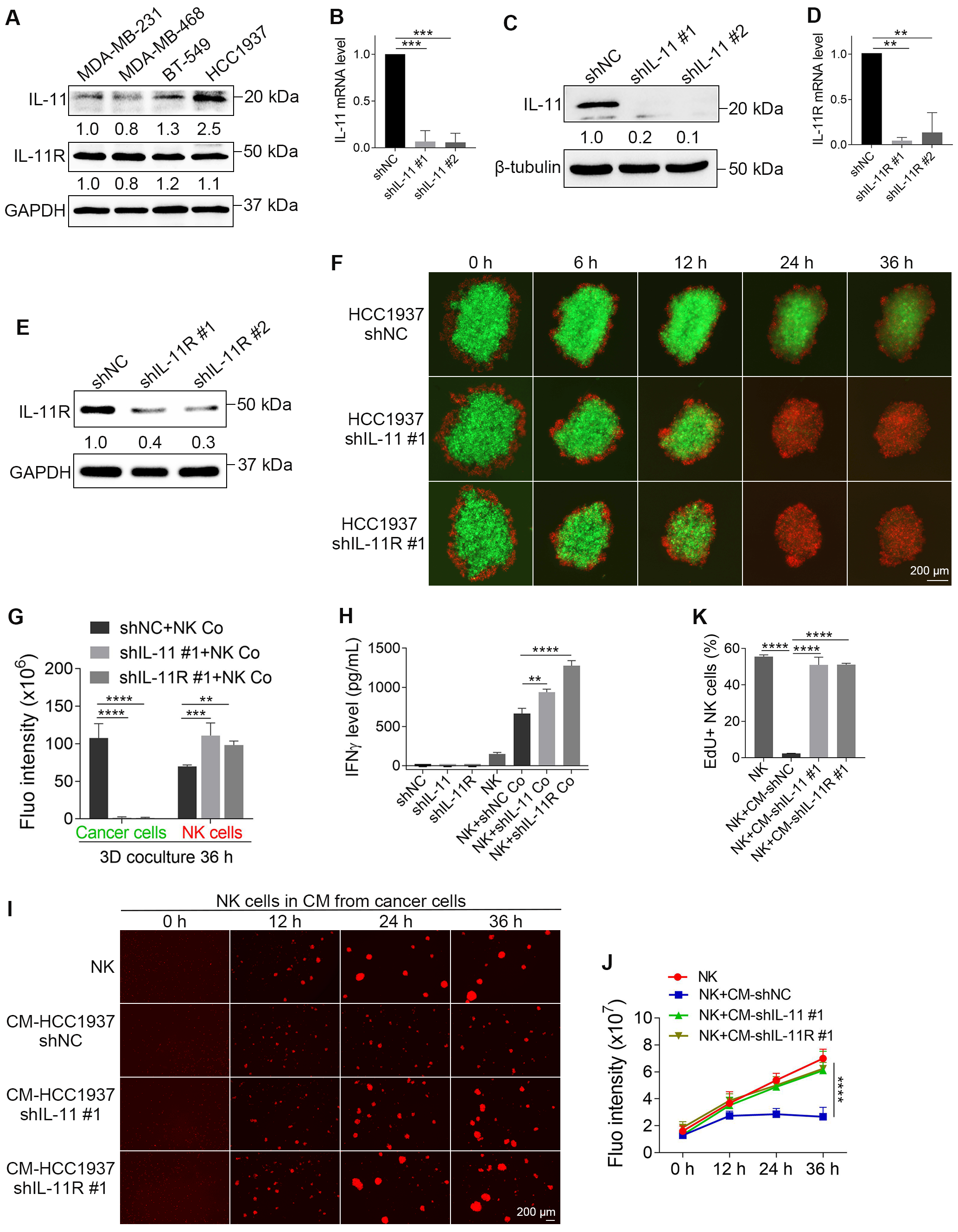


**Figure S8.** Knockdown of either IL-11 or IL-11R makes HCC1937 cells sensitive to NK cells. A) WB analysis shows the protein levels of IL-11 and IL-11R in four TNBC cell lines (n=3). B-E) qPCR and WB show the knockdown of IL-11 and IL-11R in HCC1937 cells (n=3). F) Fluorescent images show the coculture of IL-11-knockdown or IL-11R-knockdown HCC1937 cells with NK cells. G) Quantified results show the total green and red fluorescence intensities after 36 h of coculture in (F) (n=4). H) The concentrations of IFNγ after 24 h of culture (n=3). I) Fluorescent images show the proliferation of NK cells cultured in the CM from IL-11-knockdown or IL-11R-knockdown HCC1937 cells. J) Quantified results show the total fluorescence intensities of NK cells in (I) (n=3). K) The percentages of EdU-positive NK cells after being cultured for 24 h in different media (n=3). The sizes of scale bars are indicated in each image. The data were presented as mean ± SD. Statistical significance was determined by two-way ANOVA (G, J) or one-way ANONA (B, D, H, K). ***p* < 0.01, ****p* < 0.001, *****p* < 0.0001.


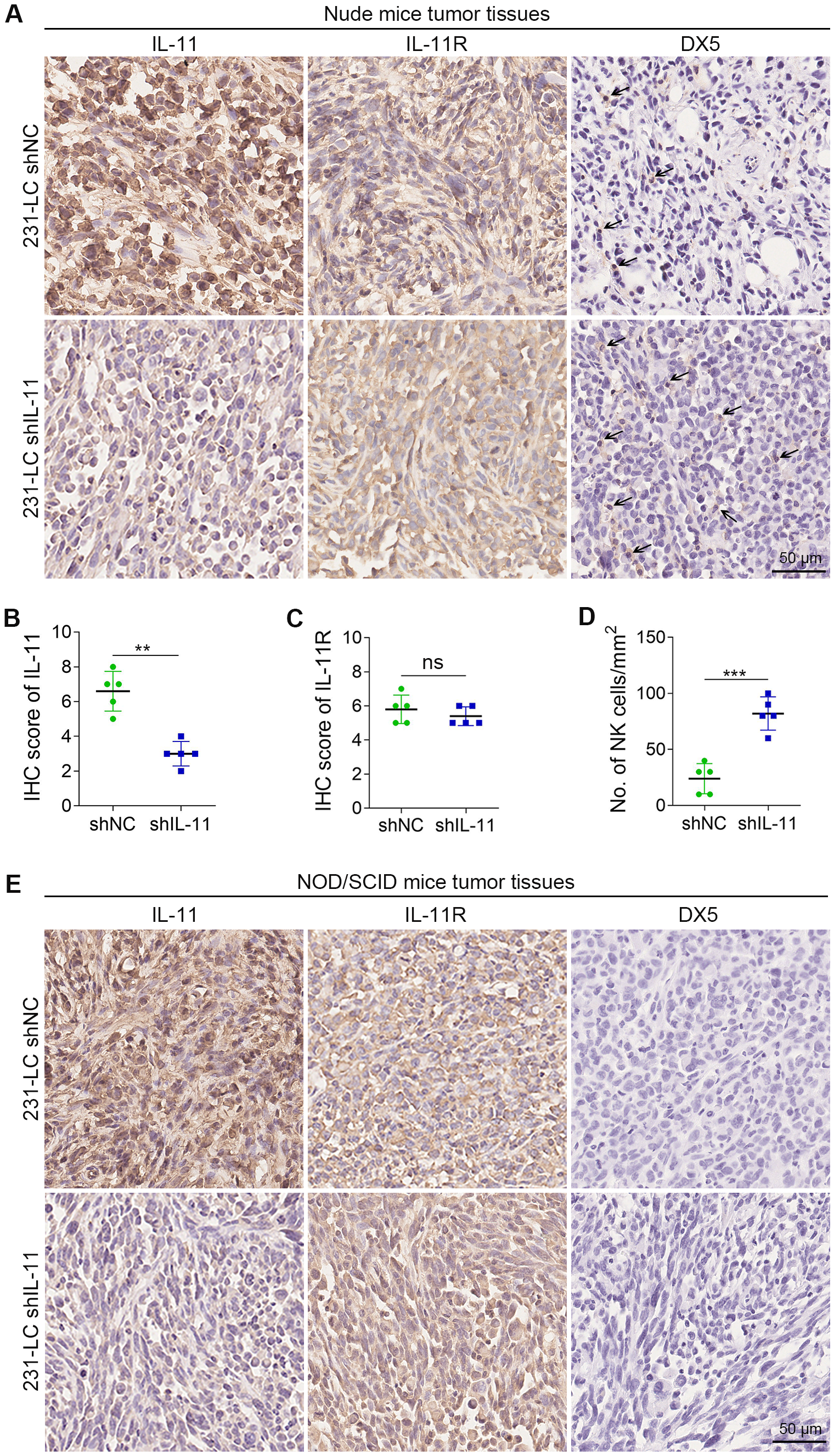


**Figure S9.** The number of NK cells in orthotopic tumors from Nude and NOD/SCID mice. A) IHC images show the staining of IL-11, IL-11R, and DX5 in control and IL-11-knockdown 231-LC orthotopic tumors from Nude mice. The NK cells are indicated with arrows. B, C) IHC scores of IL-11 and IL-11R (n=5). D) Number of NK cells in the TME (n=5). E) IHC images show the staining of IL-11, IL-11R, and DX5 in control and IL-11-knockdown 231-LC orthotopic tumors from NOD/SCID mice. The sizes of scale bars are indicated in the image. The data were presented as mean ± SD. Statistical significance was determined by *t*-test (B, C, D). ***p* < 0.01, ****p* < 0.001, ns: not significant.

**Supplementary Table 1. List of shRNAs used in this study.**

| shRNA name | Target sequence (5’-3’) |
| --- | --- |
| shIL-11#1 | CCTGACCCGCTCTCTCCTGGC |
| shIL-11#2 | CCACCTGGCCCCCCTCGAGTT |
| shIL-11R#1 | AGCATCTTGCGCCCTGACCCA |
| shIL-11R#2 | TGGAGCCAGTACCGGATTAAT |

**Supplementary Table 2. List of the primers used in this study.**

| Gene name | Direction | Sequence |
| --- | --- | --- |
| GAPDH | Forward | CTGGGCTACACTGAGCACC |
|  | Reverse | AAGTGGTCGTTGAGGGCAATG |
| IL-11 | Forward | CGGACAGGGAAGGGTTAAAG |
|  | Reverse | CAGGCGGCAAACACAGTTC |
| IL-11R | Forward | AGGTCCGTGAAGCTGTGTTG |
|  | Reverse | TCTCATAGTCGGCTGCTTGG |
| CSF3 | Forward | GCTGCTTGAGCCAACTCCATA |
|  | Reverse | GAACGCGGTACGACACCTC |
| SRGN | Forward | AGTTTCACTTCACGAGCTTGGC |
|  | Reverse | ACCCATTGGTACCTGGCTCT |

**Supplementary Table 3. List of the antibodies used in this study.**

| Antibody name | Company | Catalog # | Application |
| --- | --- | --- | --- |
| GAPDH (14C10) antibody | CST | 2118S | WB (1:1000) |
| β-tubulin antibody | CST | 2146S | WB (1:1000) |
| Caspase-3 antibody | CST | 9662S | WB (1:1000) |
| APC anti-human/mouse Granzyme B recombinant antibody | Biolegend | 372204 | FACS (1:100) |
| APC anti-human Perforin antibody | Biolegend | 353311 | FACS (1:100) |
| APC goat anti-mouse IgG antibody | Biolegend | 405308 | FACS (1:100) |
| IL-11 antibody | Santa Cruz | Sc-133084 | WB (1:500)  IP (1:80) |
| IL-11 antibody | LifeSpan BioSciences | LS-C835015 | IF (1:50)  IHC (1:100) |
| IL-11 antibody | ProSci | XP-5164 | Neutralization  (10 μg/mL) |
| gp130 antibody | Santa Cruz | Sc-376280 | WB (1:500) |
| ADAM10 antibody | Abcam | ab1997 | WB (1:1000) |
| IL-11RA antibody | Abcam | ab125015 | WB (1:1000)  IF (1:200)  IHC (1:200) |
| CD56 antibody | CST | 99746 | IHC (1:100) |
| DX5 antibody | Biolegend | 108901 | IHC (1:200) |
| Phospho-JAK1 antibody | CST | 74129S | WB (1:1000) |
| JAK1 antibody | CST | 3344S | WB (1:1000) |
| Phospho-STAT1 antibody | CST | 7649S | WB (1:1000) |
| STAT1 antibody | CST | 14994S | WB (1:1000) |
| Phospho-Stat3 (Ser727) (6E4) antibody | CST | 9136S | WB (1:1000) |
| Phospho-Stat3 (Tyr705) (D3A7) antibody | CST | 9145S | WB (1:1000) |
| Stat3 (124H6) antibody | CST | 9139S | WB (1:1000) |
| p53 (7F5) antibody | CST | 2527S | WB (1:1000) |
| p21 Waf1/Cip1 (12D1) antibody | CST | 2947S | WB (1:1000) |
| Bcl-2 (124) antibody | CST | 15071S | WB (1:1000) |
| Cyclin D3 antibody | CST | 2936S | WB (1:1000) |
| Cyclin A antibody | CST | 67955T | WB (1:1000) |
| Cyclin E antibody | CST | 20808T | WB (1:1000) |
| CDK2 antibody | CST | 18048T | WB (1:1000) |
| Mouse IgG1 Isotype Control | Biolegend | 400101 | IP (1:200)  Neutralization  (10 μg/mL) |
| Goat anti-Rabbit IgG (H+L) secondary antibody, Alexa Fluor® 594 conjugate | Thermo Fisher | A11037 | IF (1:200) |
| Goat anti-Rabbit IgG (H+L)-HRP secondary antibody | Bio-Rad | 1706515 | WB (1:5000) |
| Goat anti-Mouse IgG (H+L)-HRP secondary antibody | Bio-Rad | 1706516 | WB (1:5000) |
|  | | | |
